# Emerging biomimetic biopolymer-based composites: advancing accessible and sustainable neural disease models and therapeutics

**DOI:** 10.3389/fbioe.2025.1720254

**Published:** 2026-02-19

**Authors:** Daniela Duc, Jacob Pattem, Benjamin Gambrill, Polina Prokopovich, Aybike Kocatürkmen, Matthew Church, Amber Mays, Akash Bedi, Emmanuel Brousseau, Oommen P. Oommen

**Affiliations:** 1 School of Pharmacy and Pharmaceutical Sciences, College of Biomedical and Life Sciences, Cardiff University, Cardiff, United Kingdom; 2 School of Dentistry, College of Biomedical and Life Sciences, Cardiff University, Cardiff, United Kingdom; 3 Department of Mechanical and Medical Engineering, School of Engineering, Cardiff University, Cardiff, United Kingdom; 4 School of Engineering and Materials Science, Centre for Predictive in vitro Models, Queen Mary University of London, London, United Kingdom; 5 Faculty of Medicine and Health, University of Leeds, Leeds, United Kingdom

**Keywords:** accessibility, biomimetic biopolymer composites, neural models, neural tissue engineering, sustainability, therapeutics

## Abstract

Neurological diseases are leading causes of death globally and disability-adjusted life years (DALYs) globally. Because of this, urgency in providing technologies and essential medicines to tackle this issue is currently recognized as key in reversing this trend. Global health strategies have recognized tissue engineering as a pillar element in progressing both neurological disease research and therapy discovery. Over time, various biomaterials have been developed with a few barriers appearing along the way when considering translation for routine neurological disorders research and therapy. These barriers include accessibility, sustainability, cost-effectiveness and affordability. In this review, we discuss how biopolymers, namely biomimetic advanced biopolymers composites have emerged to answer this issue. We will explore various types of biomimetic nanocellulose-based, self-assembling peptides, glycosaminoglycan composite, advanced functionalized nanoparticles amongst others are used to create a range of innovative state-of -the-art neuronal models that can be employed for neuronal disease investigation and therapy. Finally, we will review the current factors enabling and hindering their translation and scalability (e.g. manufacturing, characterization and commercialization) and provide a Research and Development Roadmap that can be explored to facilitate their development and provision to answer the pressing global need for these technologies in positively impacting neurological disorders.

## 1 Introduction

Neurological diseases are the second leading cause of death globally and the top cause of disability-adjusted life years (DALYs), disproportionately impacting vulnerable groups such as women, the elderly, and those from low socioeconomic backgrounds (World Health Organization, 2023). If we are to reverse this trend, the World Health Organisation urgently recommends for 80% of countries to provide essential medicines and technologies for managing neurological diseases like glioma, stroke, and epilepsy, by 2031 (World Health Organization, 2023). Global health strategies, including those in the UK, emphasize the need for innovative neurotechnologies to achieve this goal. Among these, tissue engineering has emerged as a key strategy for neurological tissue repair, therapy, and progressing neurological disease research through advanced *in vitro* models (Mathieson et al., 2020; Mathieson et al., 2021).

Although biomaterial technologies for tissue engineering have advanced in recent decades, their use in neurological disease care remains limited. Key barriers include a low prioritization of neurological disorders, inadequate research investment, outdated treatment guidelines, complex regulations, weak supply chains, limited local manufacturing, a shortage of trained staff, poor pharmacovigilance, fragmented care, ethical concerns, and restricted data access—all compounded by rising global healthcare costs and system crises (World Health Organisation, 2024b). There is therefore the imperative recognition that for neural tissue engineering strategies to be successfully implemented in healthcare, these barriers must be tackled not only during the development process but also integrated into the biomaterial design itself.

To address these challenges, pristine biopolymers (e.g., cellulose, collagen, silk) have been explored for designing neural tissue engineering scaffolds. These materials have proven highly effective in creating sustainable and cost-efficient biomimetic scaffolds for neural tissue engineering therapies and *in vitro* models. They offer significant advantages, such as cost-effective sourcing, high biocompatibility, minimal immune response, and the ability to mimic the extracellular matrix (ECM). However, they also present limitations in biodegradability, mechanical and structural integrity, and manufacturing capabilities. As a result, this has led to the exploration of advanced biomimetic biopolymer composites, which retain the benefits of native biopolymers while incorporating additional properties that enhance manufacturability, scalability, mechanical strength, electrical conductivity, and controlled-drug release. These emerging composites aim to generate highly efficient, accessible, and sustainable models for neural disease research and therapy.

In this review, we will first explore the emerging biomimetic biopolymer composites used in neural tissue engineering and their physicochemical and biomimetic properties (such as nanocellulose-based, self-assembling peptides, collagen-hyaluronan composites, glycosaminoglycan composite, advanced functionalized nanoparticles amongst others) (Paviolo et al., 2020; Evans et al., 2024; Roshanbinfar et al., 2025). We will also evaluate the current state-of-the-art *in vitro* models that utilize these biomimetic biopolymer composites (organo-on-a-chip models, bioprinted models, hydrogel bases co-culture systems and brain organoids). We will also review the injectable biomimetic biopolymer materials targeting tissue regeneration in high -impact neurological disorders such as stroke, traumatic brain and spinal cord injury amongst others). Finally, we investigate the translation of such tissue engineering technologies into current healthcare, highlighting current examples. Specifically, we discuss how biomimetic biopolymer composites can address translation challenges such as manufacturing and scalability through advanced techniques while enhancing sustainability and accessibility. Additionally, this review addresses the key challenges hindering their application and propose solutions through a collaborative Research and Development Roadmap.

## 2 Overview of the anatomy and physiology of the central nervous system (CNS)

High-level engineering of organs requires clear understanding of the anatomical components and the tissular composition of the targeted organ for accurate design. The human nervous system has evolved to control the voluntary and involuntary functions of the body, perceive the environment, process complex information and control bodily responses, emotions and movement. Our nervous system comprises of two main components: the central nervous system (CNS), and the peripheral nervous system (PNS), which mainly englobes the peripheral nerves and sensory apparatuses (autonomic, enteric and somatic nervous systems) to perform autonomic regulation, sensory perception and motor control (Daniel and Care, 2020). The CNS is structurally sub-divided into the brain and spinal cord which is protected by the vertebrate axial skeleton (skull and vertebral column). They are both complex organs composed of several different types of anatomical elements, themselves constituting of different types of tissues.

### 2.1 The brain

**FIGURE 1:**
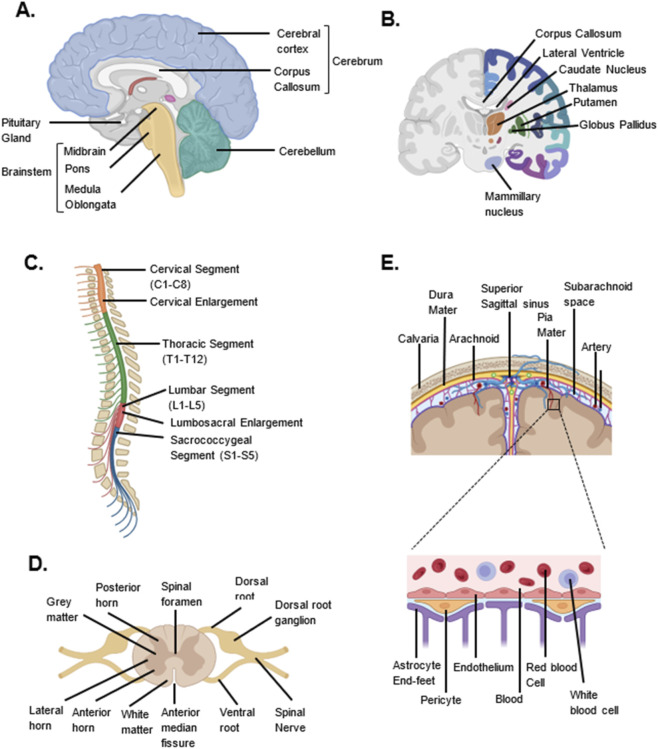
Anatomy of the Brain and Spinal Cord. **(A)** Schematic image of the main parts of the Brain: Cerebrum, Cerebellum and Brainstem (sagittal view). **(B)** Schematic illustration of the key subcortical structures of the Brain (coronal view). **(C)** Schematic image of the segments of the spinal cord (sagittal view). **(D)** Schematic illustration of the transverse section of the Spinal cord. **(E)** Schematic image of the meninges and blood-brain-barrier. Image created in Biorender.

The brain consists of the cerebrum, the cerebellum and the brainstem (Figure 1). These greatly differ in function and tissular composition. The cerebrum is composed of two hemispheres. Each are organized into 4 lobes: frontal lobe (for motor, language and cognitive functions), parietal lobe (for sensory, motor, and memory functions), temporal lobe (for emotions, language and memory function) and the occipital lobe (e.g., for vision) (Maldonado and Alsayouri, 2023) The outer layer of the cerebrum, the cerebral cortex, is highly folded and is composed of grey matter made of approximately 16 billion interconnected neuronal cells (Cadwell et al., 2019; Maldonado and Alsayouri, 2023). It is organized into the allocortex and neocortex, the latter structured in layers (I-VI) (Cadwell et al., 2019; Maldonado and Alsayouri, 2023). Its extracellular matrix (ECM) is composed of 80% water and mainly non-fibrillar proteins such as hyaluronan, aggrecan, brevican, neurocan, SPOCK/testican, phosphacan amongst others, conferring it a gel-like structure (Tallinen et al., 2016; Oros-Peusquens et al., 2019; de Jong et al., 2020; Karamanos et al., 2021).

Underneath the cortex is the white matter, essentially composed of myelinated nerve fibers with the corpus callosum being an important anatomical region interconnecting the left and right hemispheres (Maldonado and Alsayouri, 2023) (Figure 1). Glial cells, such as oligodendrocytes give rise to and maintain the myelin sheath surrounding these nerve fibers. The myelin sheath is estimated to be 35%–55% hydrated (Kister and Kister, 2022). It is composed of 70% lipids (40% cholesterol, 40% phospholipids, and 20% glycolipids) and 30% proteins (38% proteolipid protein, 30% myelin basic protein, amongst others) by dry weight (Kister and Kister, 2022). All of these structures are carefully vascularized by a complex network of blood vessels embedded within a blood-brain-barrier (BBB) specifically structured for neural tissues (Figure 1) (Reed et al., 2019). Level of alterations the BBB structure can lead to or be influenced by neurological disease states (Reed et al., 2019).

The other subcortical structures of the cerebrum include the hypothalamus, thalamus, amygdala, hippocampus, nucleus accumbens and caudate nucleus, containing the substantia nigra (Daniel and Care, 2020) (Figure 1). These structures are mainly formed of grey matter, but also contain white matter, and are organized in various nuclei (Forstmann et al., 2016; Wen et al., 2016; Whyte et al., 2024). These particular structures are subject to various genetic influences (Wen et al., 2016). Their clear structure and function are currently heavily investigated. They are especially essential in modelling and understanding disease conditions, e.g., dysfunction in dopaminergic neurons in the substantia nigra may lead to Parkinson’s disease (Wen et al., 2016; Brambrink and Kirsch, 2020; Daniel and Care, 2020).

Other components of the brain include the brain stem and the cerebellum (Figure 1). The brain stem is responsible for maintaining and controlling vital functions namely heart rate, breathing, consciousness, sleep-wake cycle and autonomic activity (Daniel and Care, 2020). It comprises of the midbrain, pons and medulla oblongata. These are also composed of grey matter (nuclei) and white matter tracts with distinct fiber organisation (Donatelli et al., 2023; Shah et al., 2020). While being the connecting point between the cerebrum and the spinal cord, it also acts as the point of emergence of several cranial nerves (Daniel and Care, 2020). The cerebellum, on the other hand, is structured into three lobes (anterior, posterior and flocculonodular). It coordinates muscular movement, posture and balance (Daniel and Care, 2020). Its highly folded cortex consists of an outer grey matter consisting of stellate, Purkinje and basket cells and an inner white matter region (Van Essen et al., 2018; Rizza et al., 2021).

### 2.2 The spinal cord

The spinal cord is an integral part of the CNS (Figure 1). It acts as a communication highway between the brain and the peripheral nervous system. It is involved in the transmission sensory input from the body to the brain, transmission of motor output from the brain to muscles (e.g. upper and lower extremities) and glands, movement coordination, autonomic functions and reflex actions (Daniel and Care, 2020; Hermes et al., 2023). This cylindrical structure, located within the vertebral canal, is continuous to the medulla oblongata. Measuring 45 cm in the length and 1 cm in width, the spinal cord extends from the foramen magnum to the second lumbar vertebra (Hermes et al., 2023). It is organized into several segments namely cervical, thoracic, lumbar and sacral (Figure 1). Several longitudinal grooves are located at its surface (anterior median fissure, anterolateral sulcus, posterior median sulcus, posterolateral sulcus). A transverse section of the spinal cord reveals an area of grey matter in the shape of an H, mainly composed of neuron bodies, surrounded by white matter (Hermes et al., 2023) (Figure 1). The grey matter is composed of projections of sensory fibers from the dorsal root ganglia (dorsal horn), spinal preganglionic autonomic neurons (lateral horn of the thoracic segments only) and motor neurons (ventral horn) (Hermes et al., 2023). The white matter is essentially composed of tracts of myelinated nerve fibers that can be ascending or descending. They are organized into the dorsal, lateral and ventral funiculi (Hermes et al., 2023).

### 2.3 CNS tissue properties in health and disease

We understand therefore that the human CNS is a complex anatomical structure composed of a combination of four main categories of tissues: neuronal tissues, vascular tissues, connective tissues and specialized cells. The neuronal tissue is itself composed of neurons and glial cells, namely astrocytes, oligodendrocytes, microglia and ependymal cells. The neuronal cells bodies are often associated with the grey matter while the neuronal axons and glial cells are often associated with the white matter. This simplified description brings us to comprehend that the extracellular matrix associated with these anatomical structures is also very specialized and greatly influences the properties of these tissues. The neural extracellular matrix can be classified as the basement membrane, perineuronal net, interstitial and perinodal matrix. When considering the mechanical properties of healthy brain tissue, the Young’s modulus is typically around 1kPa, with the grey matter often having a lower Young’s modulus than white matter. This varies with the cellular components, myelin and ECM composition (Sáez et al., 2023). Brain tissue shows viscoelastic properties with shear modulus of about 1 kPa (Budday et al., 2017). Budday et al. (2017) further found that CNS neural shear modulus further varied between 0.4–1.0 kPa for different anatomical structures (e.g. cortex, corpus callosum and basal ganglia). Neural tissue also show conductive properties ranging up to 0.6 S m-1 (Li et al., 2024). The neural extracellular matrix, in fact, is composed proteins sustaining neural tissue conductivity and bioelectricity. For instance, Susuki et al. (2013) showed the contribution of neurofascin-186 and perinodal extracellular proteins (e.g. Brain-specific hyaluronan-binding link protein)in the clustering of Na + -ion channels at the nodes of Ranvier, thus sustaining neural conductivity.

Disease states and ageing can further alter the physical and chemical properties of CNS tissues due to their influence on cellular characteristics and extracellular matrix properties (Lepelletier et al., 2017). In glioma, the brain tissue stiffness is recognized to increase from 1 kPa to 12 kPa (Niu et al., 2015). Extracellular matrix alteration is intimately linked to Alzheimer disease development and evolution. Lepelletier et al. (2017) evidenced the increase in collagen IV, perlecan and fibronectin in Alzheimer patients which are basement membrane-associated ECM molecules. Such alterations can affect the brain vascular structure and in turn influence the brain tissue structure and properties. Differences in brain tissue properties in disease states may occur due to individuals’ biological characteristics (e.g. biological sex). Finan et al. (2017), for instance, observed that in traumatic brain injury, a difference in tissue stiffness between males and female brain tissue may occur, with modest effect in grey matter but considerable effect in white matter. This may be due to differences in trauma types experienced by males and females, especially in sports (Finan et al., 2017). Such factors therefore need to be considered when accurately modelling brain tissues in health and disease.

## 3 Emerging biomimetic biopolymer composites used in neural tissue engineering

The thorough understanding of neurological disorders and the discovery of their related treatments requires precise pre-clinical investigations *in vitro* and *in vivo*. While several *in vitro* models are currently being used (as explored in Section 4), there is the recognition that there is the undisputable need for more advanced models to better replicate healthy and diseased neuronal tissues. This has motivated the development of synthetic and natural biopolymers to create these advanced *in vitro* models. With the current need for sustainability in healthcare, the widening health gap across the globe and emerging financial crises in healthcare systems, there is now a need for more affordable, accessible and sustainable technologies (Department of Health and Social Care - UK, 2025). Therefore, natural biopolymers and their advanced novel derivatives are again being heavily explored not just to answer these needs, but also due to their increased biomimetic properties.

### 3.1 Overview of natural biopolymers

The main natural biopolymers explored for biomimetic neural tissue models include cellulose, glycosaminoglycans, alginate, agarose, chitosan and collagen. The polysaccharide cellulose originate from wood, bacteria and algae (Seddiqi et al., 2021), with one of the main advantages being its abundance, and therefore low cost, alongside other biopolymer benefits of biocompatibility and biodegradability (Courtenay et al., 2018). Cellulose is formed of glucose monosaccharide units linked together by β-1,4 glycosidic bonds to form unbranched chains, which through hydrogen bonding form 3 nm microfibrils (Mutwil et al., 2008). It is not soluble in water due to hydrogen bonds enabling interactions between cellulose chains, producing complex fiber structures with high tensile strength (Zuppolini et al., 2022). To overcome poor conductivity cellulose composites are usually mixed with conductive/electroactive oligomers to improve properties desirable for successful outcomes in neural tissue engineering. Bacterial cellulose polymerized with pyrrole showed increased neuronal cell adherence via electrical simulations compared to bacterial cellulose alone (Muller et al., 2013).

Glycosaminoglycans (GAGs) are complex linear polysaccharides found ubiquitously in the brain’s extracellular matrix (ECM). Hyaluronic acid (HA), and chondroitin sulphate (CS) are the major GAGs that are found in the brain (Kwok et al., 2012) and exist as proteoglycans such as lecticans, aggrecan, versican, neurocan and phosphacan. The GAGs and proteoglycans make up approximately 20% of the brain’s extracellular matrix (ECM) and play vital roles in nervous system development. Their spatial and temporal expression patterns regulate key processes, including neurite outgrowth, precursor cell specification and maturation, as well as cell behaviors such as migration, axonal pathfinding, synaptogenesis, and synaptic plasticity (Schwartz and Domowicz, 2018). HA is widely used for CNS tissue engineering as it provides structural scaffolding that influences cell adhesion, migration, and differentiation and create specific microenvironments that can regulate neuronal outgrowth and synaptic plasticity (Jensen et al., 2020). CS proteoglycans (CSPGs) on the other hand forms perineuronal nets (PNNs) around the neurons that regulate synaptic stability and plasticity, influencing learning, memory, and critical periods of development and brain disorders (Avram et al., 2014). They can inhibit axonal regeneration, contributing to the limited repair capacity of the adult CNS. Heparan sulphate proteoglycans (HSPGs) are key components of the endothelial glycocalyx, also play an important role in neurogenesis (Yu et al., 2017) and heparin, a highly sulphated analogue of HS are grafted on hydrogels to stabilize sensitive growth factors like FGF2 for neuronal differentiation of stem cells. The HS rich glycocalyx, is vital for maintaining BBB integrity, regulating the passage of molecules and cells into the brain, and preventing cerebral oedema.

Alginate and agarose are derived from a variety seaweeds (Chudasama et al., 2021) whilst chitosan is broader in origin, being found in insects, fungi and algae (Pellis et al., 2022). Structurally alginate consists of random arrangements of α-L-glucuronic and β-D-mannuronic monosaccharides bonded by β-1,4 glycosidic bonds (Hasanzadeh et al., 2023), similar to chitosan, however with monosaccharides D-glucosamine and N-acetyl-D-glucosamine (Islam et al., 2017). Hydrogels, derived from agarose, have the advantage of self-gelling via hydrogel bonds and helix formation without the need for crosslinking agents, which could reduce biocompatibility if toxic crosslinking agents are used (Xiong et al., 2005). Chitosan is obtained by deacetylation of chitin and is cationic whereas alginate is negatively charged in nature, and as such, chitosan and alginate are used for delivery of negatively and positively charged molecules respectively (Salatin and Jelvehgari, 2017). Both chitosan and alginate have disadvantages of poor solubility in physiological pH, where solubility is possible in acidic environments, which is often reflected in methodologies which prepare chitosan (Shariatinia, 2019) and alginate (Lee and Mooney, 2012). As with cellulose, to overcome poor conductivity, oligoaniline has been used within agarose hydrogel producing a biomimetic material offering similar properties to standalone agarose, whilst improving cell adhesion to the agarose scaffold and conductivity (Li et al., 2017).

Collagen is found in connective tissue in the form of organized fibrils, providing the extracellular matrix with structural integrity (Chattopadhyay and Raines, 2014). There are 28 of which all have right-handed triple helix domains in which there are repeating three residue motif, glycine followed usually by proline and then hydroxyproline (Fidler et al., 2018; Tang et al., 2022). Collagen gels have weak mechanical properties and degrades rapidly, however, covalent crosslinking of collagen by carbodiimide chemistry or photo crosslinking by riboflavin enhance its mechanical properties and enzymatic stability, providing new opportunities in material development. Degradation of collagen can be exploited to produce collagenous materials with desirable mechanical properties for biopolymer use in biomedical applications (Adamiak and Sionkowska, 2020). Given its presence in neural basal membrane and skin dermis, collagen is biocompatible, with its use in bone implants approved by the Food and Drug Administration (FDA) over 40 years ago (Rezvani Ghomi et al., 2021). Utilizing collagen’s low immunogenicity has found a use for collagen scaffolds in the protection of cholinergic neurons and release of Nerve growth factor (NGF) into organotypic brain. This research utilized the ability of collagen to be crosslinked, by polyethylene glycol (PEG), and therefore allowing for improving stability by modifying degradation of collagen as desired (Foidl et al., 2018).

### 3.2 Biomimetic biopolymer composites for advanced neural tissue models

While natural biopolymers have shown promise (e.g. potential for low immunogenicity and biomimetics), there is the recognition that more advanced properties are needed for their stable use in *in vitro* neural models. This has led to the emergence of various biopolymer composites with novel biomimetic properties for utilisation in neural models.

#### 3.2.1 Nanocellulose-based and other polysaccharides composites

As mentioned previously, cellulose presents several advantages but also disadvantages such as poor conductivity essential for biomimetic scaffolds for neural tissue engineering (Muller et al., 2013). This led to the exploration of advanced forms of cellulose and polysaccharide composites with cutting-edge biomimetic properties to construct neural tissue engineering solutions. These include functionalized nanocellulose composites including with other nanoparticles, fucoidan composites, modified chitosan composites, amongst others (Jonsson et al., 2015; Maclean et al., 2017; Kuzmenko et al., 2018; Bordoni et al., 2020; Torresan et al., 2025). The main interest to these materials remains their high abundance in nature and projected sustainability for sourcing materials locally, especially when considering nanocellulose. This therefore offers a viable starting pathway for accessible healthcare and facilitated access to neural technologies in both high and low resources settings (World Health Organisation, 2010; World Health Organisation, 2024a; World Health Organization, 2023).

These composites offer various advantages such as customizable conductivity through chemical functionalization (Gebeyehu et al., 2022). Bordoni et al. (2020) produced 3D printed conductive nanocellulose scaffold using nanofibrillated cellulose, alginate and carbon nanotube. They were able to produce conductive scaffolds of up to 2.132 ± 0.571 S/cm to produce highly differentiated SH-SY5Y neuroblastoma *in vitro* cell models. Additionally, their high capacity for functionalization with various chemical entities offer possibility for added biomimetic effects to enable neural differentiation and guidance of neurites extensions. Kuzmenko et al. (2018) were able to bioprint tracks of blended carbon nanotubes and cellulose nanofibrils hydrogels to guide neural differentiation and axonal direction. Cheng et al. (2022) were able to produce conductive and highly porous (about 80%) chitosan-hyaluronic-gelatine scaffolds that mimicked the neural extracellular matrix by virtue of chitosan’s similar structure to glycosaminoglycans and presence of neural extracellular components hyaluronic acid and collagen (present in gelatine) (Cheng et al., 2022).

**FIGURE 2:**
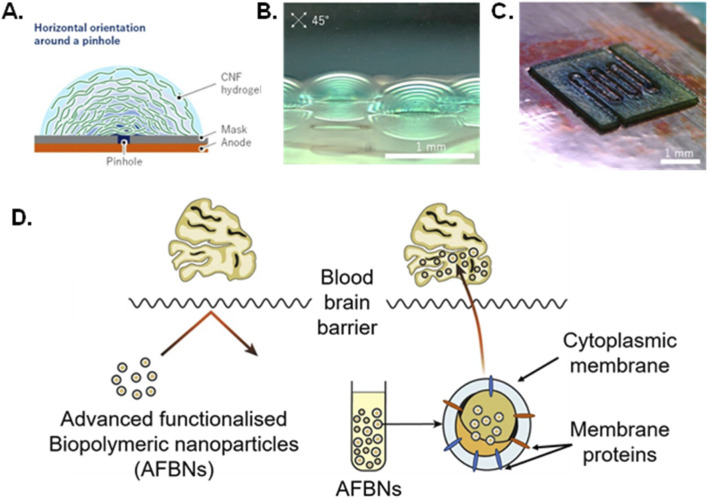
**(A,B)** The three-dimensional cellulose nanofibers oriented shape made using electrophoretic deposition and evaluated using a cross-polarizer. **(C)** a flow channel achieved using cellulose nanofibers via electrophoretic deposition Adapted (Kasuga et al., 2022) Licensed under the Creative Commons Attribution 4.0 International License (CC BY 4.0): https://creativecommons.org/licenses/by/4.0/ **(D)**. Encapsulation of core nanoparticles into cytoplasmic membrane to produce biomimetic biopolymeric nanoparticles suitable for crossing the blood brain barrier. (Adapted from Boni et al., 2018). Licensed under the Creative Commons Attribution 4.0 International License (CC BY 4.0): https://creativecommons.org/licenses/by/4.0/.

Nanocellulose and other polysaccharide composites also offer the advantage of being adaptable to several manufacturing techniques, such as electrophoretic deposition or 3D-bioprinting, making them viable for upscaling or creating various porosities and geometries for the construction of advanced scaffolds and for mechanical biomimicry (Bordoni et al., 2020; Kasuga et al., 2022). Kasuga et al. (2022) were able to produce several high throughput and standardized porous nanocellulose hydrogel scaffold of adaptable geometries using electrophoretic deposition (Figures 2A–C). While these advanced composites show promise in tissue engineering, further research is needed to further expand their properties to generate high level active biomaterials and resolve some of their limitations with certain commercial bioassays (Bordoni et al., 2020).

#### 3.2.2 Glycosaminoglycan-based hydrogels and soft composite materials

Based on the aforementioned properties of glycosaminoglycans (GAGs) seen in Section 3.1, GAG based soft hydrogels, and composite materials are extensively used for neural tissue engineering, *in vitro* modelling of brain diseases and as a reservoir for delivering stem cells, and therapeutic biologics. Induced pluripotent stem cells (iPSC) and embryonic stem cell derived neuronal progenitor cells (NPCs) are valuable sources of stem cells that are utilized for neuronal tissue engineering. Several studies also used murine spinal cord slide cultures (SCSC) and dorsal root ganglion cultures (DRGCs) to study the effect of hydrogel scaffolds on tissue regeneration.

HA-based composite gels are engineered for axonal regeneration and neuronal tissue engineering. In a recent study, axonal outgrowth from SCSC and DRGCs was evaluated in HA gels (physically and chemically crosslinked HA gels) and collagen gels. HA based hydrogels were superior for neuronal survival and neuronal protection in SCSC (Schizas et al., 2014). Superior axonal outgrowth in SCSC and DRGSs clearly indicates preferential response to HA based gels that are naturally present in the brain ECM, when compared with collagen gels (Bajic et al., 2024). Compared to culturing murine spinal cord section, culturing human iPSC and embryonic stem cell derived NPCs are challenging as they are sensitive to biophysical cues (e.g. stiffness) provided by the matrix. It was reported that the chemical cues provided by HA and CS composite gels are not sufficient for the differentiation of NPCs to neurons, however, covalent grafting of dopamine moiety traps the cell derived factors like that support neuronal differentiation (Samanta et al., 2022). Conversely, a separate study demonstrated that HA-PEG gels crosslinked via thiol-maleimide chemistry were sufficient to induce neuronal differentiation of ESC-derived NPCs (Seidlits et al., 2019), apparently contradicting the necessity of the catechol modification and CS for neuronal 3D cell culture. The discrepancy is likely due to varying stem cell protocols, such as the use of bFGF/EGF versus neurotrophic growth factors, which makes direct comparison of the matrix effects difficult. Furthermore, differences in the hydrogel’s mechanical properties (stiffness) add another layer of complication when comparing studies (Seidlits et al., 2010).

In another study, “amyloid-like” matrix was designed by crosslinking HAMA with collagen and diphenylalanine compound coupled with fatty acid chain (myr-FF) that self-assemble into a hydrogel with β-sheets and amyloid-like aggregates driven by π−π stacking interactions (Mathes et al., 2024). Human iPSC derived NPCs displayed higher levels of neuroinflammation and apoptosis markers when NPCs were cultured in the amyloid-like matrix compared to a natural brain matrix. HA-based hydrogels were engineered to study how viscoelasticity influences human spinal cord organoids (hSCOs) cocultured with blood vessel organoids (hBVOs). Hydrogels with matched stiffness but varying viscoelasticity—and vice versa—were created by crosslinking HAMA with PEG-thiol. Viscoelastic gels enhanced regional hSCO patterning, promoted dorsal marker expression, and supported interneuron differentiation more effectively than purely elastic or stiffer gels. In coculture settings, viscoelasticity had a stronger impact on organoid development than stiffness (Chen et al., 2024).

CS based hydrogels (Karumbaiah et al., 2015) and heparan sulfate-based hydrogels (Latchoumane et al., 2022) are also used for neural tissue engineering as they bind and potentiate neurotrophic factors. These GAG-based gels provide an ideal stem cell niche for promoting NSC self-renewal and neuronal regeneration. CS-derived gels encapsulated with fibroblast growth factor (FGF2) was successfully harnessed to repair and regenerate brain tissue after traumatic brain injury (Betancur et al., 2017). Hydrogel fabricated by crosslinking HA and heparin was used as a favorable environment for delivering neural progenitor cells into the infarct cavity after stroke (Zhong et al., 2010). Compared to direct stem cell injection without hydrogel support, the hydrogel mediated delivery of stem cells enhanced stem cell survival and diminished inflammation and cell stress. However, most ES- derived NPCs remained in an undifferentiated state with or without hydrogel.

#### 3.2.3 Injectable biopolymer-based composite materials for treating stroke, traumatic brain injury, brain tumors

Neurological disorders such as Traumatic Brain Injury (TBI), stroke, and brain tumors are among the leading causes of death and long-term disability across the globe (Turner et al., 2021; Cao et al., 2025). Despite differing in origins, each condition shares common pathological features including neuroinflammation, oxidative stress, disrupted neural networks, and challenges in delivering therapeutic agents across the blood-brain barrier (BBB) (Banks et al., 2024). TBI typically results from external mechanical forces, stroke from a disrupted cerebral blood flow, and brain tumors from excessive and abnormal cell proliferation (Azzarelli et al., 2018; Lafta and Sbahi, 2023; Lui et al., 2025) Traditional treatments often fall short due to limited efficacy and poor prolonged localization to the injury or tumor site (Maas et al., 2022). In recent years, injectable biopolymer-based materials have emerged as promising therapeutic platforms across all three condition examples (Aqel et al., 2023). Such materials offer minimally invasive, highly localized, and sustained delivery of therapeutics with significant potential to support tissue repair, modulate inflammation, direct the differentiation of stem cell fate, and inhibit reactive oxygen species (ROS) activity (Lu et al., 2024; Shi et al., 2025).

##### 3.2.3.1 Traumatic brain injury

One major challenge in neural tissue engineering is suppressing astrocyte differentiation while promoting neuronal differentiation of stem cells (SCs) to enhance neurorepair (Rybachuk et al., 2023; Liu X. et al., 2023) addressed this by employing an injectable hyaluronan–collagen hydrogel, loaded with bone marrow SC-derived exosomes in an *in vivo* model. This system provided a supportive microenvironment found to inhibit astrocyte differentiation and promote endogenous neural progenitor cell (NPC) differentiation into functional neurons and oligodendrocytes. Endogenous healing following TBI is often hindered by sustained inflammation, which amplifies neuronal damage (Schimmel et al., 2017). Although various anti-inflammatory drugs are available, maintaining their localized and sustained delivery remains a challenge. Macks et al. (2022) demonstrated that an injectable poly (ethylene glycol)-bis(acryloyloxy acetate)-based hydrogel loaded with dexamethasone provided sustained steroid release in an *in vivo* model, pro-inflammatory cytokines which are IL1β, TGFβ1, TNFα, and IFN*γ* were downregulated by more than 2-fold, indicating a significantly reducing inflammation, significantly reducing inflammation and enhancing neuronal repair. Reactive oxygen species (ROS) play a key role in exacerbating secondary injury following TBI and contribute significantly to neuronal death. To counter this, an injectable hydrogel composed of poly (propylene sulfide) and triglycerol monostearate, encapsulating curcumin has been proven to successfully to reduce ROS expression resulting in more than 2-fold reduction in brain oedema, thereby inducing significant neuroprotective effects (Qian et al., 2021).

##### 3.2.3.2 Stroke

As a method of enabling prolonged and targeted treatment of ischemic strokes, Gu et al. (2023) developed an injectable hyaluronic acid-based hydrogel loaded with exosomes derived from neural progenitor cells (NPCs). When implanted into a mouse model of middle cerebral artery occlusion, the hydrogel significantly improved neural recovery, particularly motor function, while also promoting angiogenesis and reducing inflammation. Similarly, in an effort to mitigate the inflammatory response associated with stroke, Guo et al. (2025), designed a gelatine and oxidized dextran (ODex) hydrogel capable of sustained nitric oxide (NO) and ISO-1 release. In a comparable mouse stroke model, treatment with this hydrogel led to marked reductions in pro-inflammatory cytokines IL-6 and IL-10, along with enhanced angiogenesis.

##### 3.2.3.3 Brain tumors

Injectable biopolymers have been identified as a potential method of treatment for brain tumors alongside TBI and strokes, due to conventional therapies such as radiotherapy and chemotherapy having renowned limitations. To enhance local drug delivery and overcome the limitations of systemic chemotherapy, thermosensitive copolymers can act as injectable drug carriers, allowing *in situ* gelation for glioblastoma treatment. One study combined two thermosensitive polymers, Pluronic F127 (PEO-PPO-PEO) and PLGA-PEG-PLGA, to deliver salinomycin directly to the tumor site. By remaining liquid at cold temperatures, but forming a gel at 37 °C, the authors Norouzi et al. (2021) were able to establish a drug depot within the target region to provide sustained and localized release.

#### 3.2.4 Self-assembling peptides and other peptide biopolymer composites

Self-assembling peptides (SAPs) represent a versatile class of biomimetic biopolymers with growing relevance in neural tissue regeneration disease modeling, treatment diagnostics, antimicrobials, cell culture and therapeutic delivery (Peressotti et al., 2021; Najafi et al., 2024; Cui and Tirrell, 2025). Their intrinsic ability to form well-defined nanostructures under physiological conditions, combined with bio-functionality), biocompatibility, and tunable physicochemical properties make them particularly attractive in neuroscience research (Tsai et al., 2025; Wychowaniec et al., 2025). SAPs are composed of short, repetitive amino acid sequences that exploit non-covalent interactions (e.g., hydrogen bonding, electrostatic forces, hydrophobic effects) to self-organize into nanostructures such as nanofibers, nanotubes, and hydrogels (Raspa et al., 2021).

Several SAP-based scaffolds have shown promise in neural contexts. Amyloid nanofibrils are β-sheet-rich assemblies that structurally mimic the native extracellular matrix (ECM), thereby supporting tissue regeneration (Das et al., 2016). For instance, RADA16 forms β-sheet nanofibers with neural tissue-like stiffness; and is used in models of brain ischemia and repair (Liu X. et al., 2022; Sun et al., 2025). A truncated version of RADA16, RADA4 has been shown to support microglial compatibility and scaffold integration (Francis et al., 2016; Sahab Negah et al., 2017). BMHP1, is a bone marrow-derived peptide featuring ECM-binding motifs, that enhance neural cell adhesion and survival (Silva et al., 2013).Finally, Rhein, a plant-derived compound that binds TLR4 in microglia, reportedly reduces neuroinflammation (Liu H. et al., 2023).

SAPs are increasingly used to mimic the neural ECM facilitating stem cell viability, engraftment and differentiation (Yaguchi et al., 2021; Forouharshad et al., 2023). Moreover, SAP-based platforms support the localized delivery of therapeutic agents, including CRISPR/Cas9 systems, gene-based therapies, anti-inflammatory drugs, trophic factors and stem cells addressing challenges in regenerative neurology (Abdolahi et al., 2021; Raspa et al., 2021). Their fibrillar nature also confers hemostatic properties, useful in neurosurgical contexts (e.g., preventing bleeding without pressure, cauterization or other potentially physically damaging techniques, whilst providing amino acids to assist with injury repair), and several SAPs support long-term primary neuron cultures, enabling more physiologically relevant disease models (Martin et al., 2018; Yang et al., 2018).

To overcome inherent limitations, such as mechanical fragility and rapid degradation biopolymer composites have been developed (Ciulla et al., 2024; Sun et al., 2025). Combining SAPs with natural or synthetic materials (e.g., collagen, silk fibroin, alginate) improves mechanical strength, degradation kinetics and bioactivity (Sun et al., 2021a; Xiao and Horng, 2025). Hybrid scaffolds like micropatterned silk-SAP composites have demonstrated enhanced neuronal differentiation (Bai et al., 2014; Sun et al., 2021b). Crosslinking agents (e.g., genipin, transglutaminase) further modulate scaffold stiffness and longevity (Ciulla et al., 2024). Moreover, integrating electroconductive elements (e.g., polyaniline, tetraaniline, Polyvinylidene fluoride) enables scaffolds to mimic neural electrical signaling, improving tissue interfacing and functional integration (Forouharshad et al., 2024).

While SAPs offer a compelling platform for neural tissue modelling and therapy, their full potential lies in hybrid/functionalized systems e.g., aniline/conductive polymer-SAPs, collagen mimetic self-assembling peptides, functionalized SAPs, SAPs with natural ECM components such as laminin (Marchini et al., 2020; Sun et al., 2025). By combining SAPs with functional biomaterials and bioactive modifications researchers can engineer next-generation scaffolds that closely replicate the native ECM, support regenerative processes and enable targeted, precision therapies in neurodegenerative and traumatic CNS conditions (Tran et al., 2020; Ohno et al., 2023).

Both Tran et al. (2020) and Ohno et al. (2023) illustrate how restoring protective barriers and providing cell-instructive cues can be leveraged to address region-specific challenges in central nervous system regeneration. The RADA-16I scaffold developed by Tran and co-workers facilitates the restoration of microvascular integrity, thereby limiting inflammation and glial scarring while enhancing axonal ingrowth. In contrast, the Ncad-mRADA hydrogel designed by Ohno and colleagues provides adhesive guidance cues that recruit and direct endogenous neuroblasts to sites of brain injury, promoting neuronal replacement and circuit formation. These strategic differences are reflected in their experimental models, with RADA-16I evaluated in a rat spinal cord contusion model and Ncad-mRADA tested in deep brain and neocortical injury paradigms.

#### 3.2.5 Advanced functionalized biopolymer nanoparticles

Biopolymers show potential for uses in neural tissue engineering as they are biocompatible and biodegradable, resemble components of extracellular matrices, and provide a scaffolds (Kruczkowska et al., 2024; Orive and Desimone, 2024). Nanoparticles have the advantage of being modifiable to make them biomimetic, and can be designed to mimic local environments whilst functioning in a desired manner (Zinger et al., 2021). Biomimetic nanoparticles usually consist of a biopolymeric core surrounded by cytoplasmic membrane with membrane proteins (Figure 2D), where biomimetic nanoparticles pass across blood brain barriers, allowing for delivery of advanced functionalized biopolymer nanoparticles in neural applications (Boni et al., 2018; Tripathi et al., 2022; Zhong et al., 2023).

Both chitosan microspheres (≤1,000 mm) and nanoparticles (≥100 nm) have been prepared with molecules to target central nervous system (CNS) and spinal cord injuries (SCI). When chitosan nanoparticles were encapsulated into the alginate/polypyrrole (Alg-PPy) scaffolds the scaffold could attach to neural cells and enable proliferation (Azizian et al., 2018; Manzari-Tavakoli et al., 2020). Alg-PPy scaffolds with nanochitosan have been shown to be capable of electrical conductivity, a requirement for the nervous system and encouraging nerve tissue regeneration (Manousiouthakis et al., 2022). As such there is much interest in conductive hydrophilic polymers, such as PPy, polyphenylene and polythiophene (Das and Prusty, 2012), where electron transfer occurs across the conjugated polymer matrix (Ramesan and Suhailath, 2017). Inclusion of alginate with PPy provides an interactive substrate for further electrical interactions using positively charged PPy and the carboxylate groups of alginate (Lin et al., 2019). The addition of nanochitosan into the scaffold increases both the surface area and hydrophilicity, making the scaffold more suitable as a biomimetic material for neural cell attachment. A ratio 2:10 PPy-Alg was shown to have sufficient conductivity for neural cell attachment and proliferation (Manzari-Tavakoli et al., 2020), demonstrating nanochitosan-Alg-PPy constructs offer a suitable platform for neural tissue support and, or engineering. Following laminectomy and injection of PPy-Alg-nanochitosan into Wistar rats showing reduction of inflammation, allowing for neural regeneration to occur (Manzari-Tavakoli et al., 2024). Similarly, in Sprague-Dawley rats, PPy-Alg-carboxymethyl chitosan was injected subcutaneously after which only basal inflammatory responses were observed despite hydrogel infiltration into adjacent tissue, with no severe responses observed (Bu et al., 2018).

Biopolymer nanoparticles can also be used for encapsulation of important compounds to produce drug carriers which could then be incorporated into scaffolds (Hudson and Margaritis, 2014). Brain derived neurotropic factor (BDNF), which has a protective role following neuronal injury, was used in conjunction with chitosan nanoparticles, independently and plasmid DNA encoding BDNF (Lopes et al., 2017; Mahanta et al., 2024). In both cases the chitosan nanoparticles were modified for specific targeting; Chitosan nanoparticles were grafted with tetanus neurotoxin to target peripheral neurons (Lopes et al., 2017), and modified with mannose to target glucose transporter-1 on the blood brain barrier (Mahanta et al., 2024). In nerve crushed mice BDNF plasmid DNA was injected and those mice showed decreased muscle atrophy, earlier reinnervation compared with control groups in line with increased BDNF expression, and correlating with nerve protection (Lopes et al., 2017). In testing nanoparticles produced by Mahanta et al. (2024) against blood from Sprague–Dawley rats, their research found 200 μg/mL nanoparticles having a hemolysis percentage of 10%, above which would be considered hemotoxic and unsuitable for delivery systems for treatments addressing Alzheimer’s disease (AD) (Yedgar et al., 2022). Nerve growth factor (NGF), a requirement for axonal regeneration and neurotransmission was encapsulated into chitosan nanoparticles using tripolyphosphate crosslinker, where resulting nanoparticles could differentiate stem cells into neurons (Mili et al., 2018). Gold nanoparticles, and chitosan nanoparticles encapsulated with BDNF, which were then suspended in laminin coated PLGA fibers, resulted in regenerated and repaired adipose-derived stem cells, simulating peripheral nerve injury (Jahromi et al., 2021). Similarly, chitosan nanoparticles were encapsulated with gold and NGF (Razavi et al., 2019). It has been shown that gold nanoparticles provide sites for neuronal cell attachment, subsequent differentiation, growth and maturation (Baranes et al., 2016), including via electrical excitation (Paviolo and Stoddart, 2017). Gold nanoparticles have also been used in gelatin hydrogels where electrical conductivity was enabled with parahydroxybenzaldehyde (Barabadi et al., 2024), suggesting further opportunities using gold nanoparticles for electrical conductivity within a hydrogel substrate. A hydrogel combination of chitosan and alginate encapsulated with berberine (positive acting towards Alzheimer’s disease) resulted in regeneration of sciatic nerve in the berberine hydrogel group but also the hydrogel mixture only group (Rahmati et al., 2021).

To further the potential of biodegradable nanocomposite scaffolds, novel biopolymers are being developed. Polycondensation of glycerol and sebacic acid, followed by crosslinking produces polyglycerol sebacate (PGS), a biodegradable and biocompatible polymer (Godinho et al., 2022), with similar elasticity to peripheral nerves, as determined using Young’s modulus (Rai et al., 2012). Functionality of PGS was improved by copolymerizing PGS with poly-ε-caprolactone (PCL) via electrospinning to produce fibers within a scaffold, to which hydroxyapatite (HA) nanoparticles were incorporated along the fibers. The final material provided a substrate for tissue repair studies, with increased surface area for cell attachment, support and proliferation along the fiber structures (Pourhoseyini et al., 2024). Increasing incorporated HA nanoparticles resulted in greater cell viability of PC12 cells using MTT assay, and cell adhesion onto the scaffolds due to their suitable surface hydrophilicity (Saudi et al., 2022).

## 4 State-of-the-art *in vitro* models for central nervous system – opportunities and challenges

Research on neurological disorders are extremely important for understanding underlying disease pathophysiology and discovering new therapies. Such research is highly dependent on research tools, especially *in vitro* neural models, to help investigate disease and inform therapy development. Choice of *in vitro* models for neurological disorders investigations especially are dependent on researcher’s capability, availability of the model, equipment, reliability of the model, its comparability and standardization. Due to these key factors, routine use of biomaterials-supported advanced neural cell models in standard practice of neurological disease investigation, is still under development.

### 4.1 2D engineered neural models

2D cell culture models are the simplest types of cell culture models used for neurological disease drug testing, disease exploration and to produce cell therapies. They are usually created on tissue culture plastics, and differentiation, growth or proliferation are induced either via signaling molecules included in the tissue culture media or genetic means. They are often used for their simplicity of *in vitro* cell culture, standardization, broad commercial availability, relative cost-effectiveness, inter-comparability and relative reliability. This in spite of their limitation in terms of ability to exactly mirror *in vivo* normal or disease conditions. Recently, biomaterials, including biomimetic biopolymers, have been used to create advanced 2D neural cell culture models with and without the mediation of signaling molecules. While these are not common in everyday laboratory practice for neurological disease research yet, these have shown great potential.

For instance, Kaur and Roy (2021) created an advanced 2D neural cell models, using primary pentapeptide sequence of the N-cadherin protein to mimic the neural extracellular matrix (ECM) to enhance cell-cell interaction mediated by ECM proteins. They found that growth of 2D cell lines within peptidic N-cadherin biomimetic scaffold produced C6 glioma 2D cell models and mouse fibroblast cell line L929 with enhance cell viability, neurite extension and cell proliferation. This could be explained by the encouragement of cell-cell interaction by the biomimetic N-cadherin peptide in the 2D scaffold (Kaur and Roy, 2021). Additionally, Mathes et al. (2024), aimed to recreated the microenvironment of Alzheimer’s Disease by the use of a self-assembling peptide-based hydrogel scaffold made of hyaluronic acid -collagen hydrogel and diphenylalanine compound coupled to a myristic acid (myr) fatty acid chain to mimic an amyloid-like matrix. They found that when incorporating neural progenitor cells in the matrix, showed a increased level of neuroinflammation and apoptosis markers in comparison with NPC grown on 2D Matrigel (Mathes et al., 2024). While this scaffold produce more 3D-like cell culture model, it indicates the potential of biomimetic scaffold in producing higher level single cell culture models that can be used routinely to better inform on neurological diseases.

Others have also explored biomimetic natural polymers for 2D co-culture neural models and to modulate neural cell behaviour to produce higher performing neural cells *in vitro*. Gonçalves-Pimentel et al. (2018) explored cellulose exopolysaccharide from sugarcane molasses as a low-cost base materials for creating astrocyte and neuronal co-cultures. They were able to show high level of astrocyte-neural co-culture viability, proliferation and activity through enhanced levels of NMDA-induced Ca2+ influx. Pandanaboina et al. (2021) further created nanocellulose substrate functionalized with lysine molecules which were able to enhance the differentiation of neural stem cells into functional neural cells thus showing its potential in producing highly viable neuronal cultures and also the potential of the biomimetic materials in NSC transplantation for therapy (Pandanaboina et al., 2021).

### 4.2 3D/4D engineered neural models

#### 4.2.1 Hydrogel based co-culture systems

Three-dimensional (3D) hydrogels are increasingly used to model complex cell–cell and cell–ECM interactions that are difficult to replicate in traditional 2D cultures or animal models. The choice of polymeric material used to engineer these hydrogel scaffolds profoundly influences cellular behavior, as cells dynamically respond to biochemical and mechanical cues from ECM-mimetic environments. The human brain ECM is largely devoid of fibrous proteins like collagen and fibronectin (which are mainly confined to the basement membrane) (Evans et al., 2024). Hyaluronic acid (HA) and chondroitin sulfate (CS) are the key components of the brain ECM and 3D scaffolds derived from HA and CS are most suitable for culturing neuronal cells. This section focuses on hydrogel-based *in vitro* disease models designed to investigate glioblastoma (GBM) and neural tissue engineering.

##### 4.2.1.1 Hydrogel coculture system for studying glioblastoma

Among different brain diseases, glioblastoma (GBM) is one of the most studied diseases using 3D hydrogel models as investigating complex cellular interactions between, immune cells, stromal cells and glioblastoma is not easy using 2D cell culture or animal models. HyStem-HP, a hyaluronic acid–gelatin composite hydrogel crosslinked with PEG-diacrylate, has been used to study GBM–microenvironment interactions. In one study, coculture of microglia and glioma cells within this matrix led to a 17%–30% increase in GBM cell proliferation and significantly enhanced drug resistance and migration, highlighting the influence of non-neoplastic cells on chemoresistance (Leite et al., 2020). In a related study, astrocyte–GBM interactions within the same hydrogel revealed that direct astrocyte–tumor contact promoted GBM growth, migration, and resistance to chemotherapy (Civita et al., 2019).

Gelatin methacrylate (GelMA) based hydrogels are the most common hydrogel matrix used to develop *in vitro* models. GelMA gels were used to study the role of microglia on GBM progression. This study aimed to decouple direct cell contact from paracrine effects between microglia and GBM cells as this model permitted soluble signaling but blocked direct cell contact. This study demonstrated that factors produced by GBM-microglia crosstalk activated microglia cells. GBM12 cells cultured within GelMA gels in close proximity to microglia cells displayed significant GBM12 cell proliferation and inhibited invasion in response to factors produced by microglia (Chen J.-W. E. et al., 2020). In another study, GelMA was blended with hyaluronic acid methacrylate (HAMA) to form a composite matrix for investigating pericyte-induced drug resistance. Coculture of pericytes with GBM spheroids led to a 160-fold increase in CCL5 expression following temozolomide (TMZ) treatment, indicating activation of the CCL5–CCR5 paracrine axis and contributing to TMZ resistance (Maity et al., 2025). Another study investigated the GBM–neuronal interactions by embedding GBM spheroids within a preformed neuronal network in Collagen-I gels. The study revealed a bidirectional paracrine communication that supports GBM progression, potentially via the phosphatidylinositol 3-kinase (PI3K)/Akt/rapamycin-sensitive mTOR-complex (mTOR) pathway (Förster et al., 2025).

Apart from gelatin- and collagen-based gels, other natural polymers such as hyaluronic acid, alginate, chitosan, and PEG-based hydrogels are also utilized to engineer biomimetic tumor models (Ahmed, 2023). However, scaffolds derived from the extracellular matrix (ECM) offer superior biomimicry, as tumor cells cultured within them secrete matrix metalloproteinases and plasminogen activators that degrade the ECM-mimetic matrix. This degradation facilitates cell invasion into the surrounding scaffold, closely replicating natural tumor metastasis and invasion processes (Kaphle et al., 2019).

##### 4.2.1.2 Hydrogels based coculture system for neural tissue engineering and brain modeling

Astrocytes and microglial cells are key regulators of neuroinflammation, and glial scarring associated with neurodegenerative diseases and traumatic brain injuries (TBI). Brain-mimetic hydrogels that support the coculture of neuronal and glial cells—such as astrocytes and microglia—offer valuable insight into neuron–glia interactions that cannot be captured by traditional 2D cultures or animal models.

Kim et al. developed a 3D neurosupportive culture platform using an RGD-functionalized, alginate-based microfiber system embedded with astrocytes. This system promotes neurite outgrowth, guided neurite directionality, and enhanced synapse formation—all without direct physical contact between astrocytes and neurons (Kim et al., 2020). Malheiro and colleagues established an *in vitro* peripheral nerve model by culturing rat primary Schwann cells (SCs) on an electrospun scaffold, combined with either primed PC12 cells or rat dorsal root ganglion (DRG) neurons embedded in a fibrin gel. Both models demonstrated aligned neurite outgrowth and formation of mature myelin segments. This system offers a cost-effective platform for drug screening on peripheral nerve-like tissue under both normal and pathological conditions (Malheiro et al., 2020). Models of traumatic brain injury and glial scarring have also been developed using cocultures of neurons with astrocytes and microglia. For example, Spencer et al. created an *in vitro* glial scar model using mixed primary embryonic neural cell cultures embedded in collagen-I gels. By applying micromechanical stimulation via high-precision linear actuators, they mimicked axial micromotion around neural implants. This resulted in localized astrocyte accumulation around stress zones, as compared to static control conditions (Spencer et al., 2017; Basit et al., 2023).

Photo-crosslinked silk fibroin hydrogels functionalized with RGD peptides and encapsulated with neural progenitor cells (NPCs) have also shown promise. When implanted into injured spinal cords, these hydrogels stimulated neurite extension, suppressed inflammation through M2 macrophage recruitment, reduced glial scar formation, and accelerated corticospinal tract axon regrowth—ultimately enhancing locomotor recovery (Ling et al., 2023). In another study, coculturing bone mesenchymal stem cells (BMSCs) and neural stem cells (NSCs) in a GelMA hydrogel enhanced NSC survival, proliferation, and differentiation into neurons, while suppressing astrocyte formation. Upon implantation into a rat spinal cord hemisection model, this system promoted axonal regeneration, improved motor function, and significantly reduced glial and fibrotic scarring as well as inflammation (Zhou et al., 2020). GelMA hydrogels have also been used to deliver iPSC-derived NSCs (iNSCs) for spinal cord injury (SCI) treatment. *In vitro*, iNSCs cultured in GelMA exhibited robust neurite outgrowth and neuronal differentiation. *In vivo* transplantation of iNSCs encapsulated in GelMA suppressed neuroinflammation, inhibited glial scar formation, and promoted axonal regeneration (Fan et al., 2018).

#### 4.2.2 Advanced brain organoids

Brain organoids are another type of 3D neural models that is currently heavily explored for neurological disorder research. They are often derived from pluripotent stem cells, with the first human-derived neural organoids being reported in 2011 (Eiraku et al., 2011). Neural organoids offer the advantage of self-organisation of various brain tissues and cells that can be often compared to early neural and brain development. They can also be derived from patients cells (e.g. via induced pluripotent stem cells technology) to replicate patient neural characteristics. At current, the culture of brain and neural organoids is often challenging as they require high-level of training, costly consumables (e.g. supplementations) and equipment for cell culture growth (e.g. when grown in suspension) and scalability. Other issues include neural organoid unpredictability, even when using standard protocols making comparability difficult, cell death due to nutrient diffusion issues and existing ethical challenges especially when looking at various level of human brain development that may be generated by current routine techniques (Gordon et al., 2021; Kataoka et al., 2024).

To answer to some of these challenges the use of advanced biomimetic biopolymers materials are currently being explored. Ferraro et al. (2025) attempted to address the issue of expertise, scalability and unpredictability of brain organoids through the use of a bioink containing Matrigel®, sodium alginate and sodium carboxymethylcellulose to create neural embryoid bodies. Through this solution, they were able to create consistent cortical organoids from induced pluripotent stem cells due to adequate mechanical factors (e.g. viscosity and elasticity) of the biomaterial. Adequate neural cell organisation and differentiation was also observed (e.g. neural rosettes and biomarkers expression) To attempt to address the issue of nutrients provision and brain vascularization *in vitro* modelling, Zhu et al. (2021) explored an alginate-carboxymethylcellulose materials and microfluidics. Their technique created consistent vascularized brain organoids via the generation of microcapsules with iPSC derived neural cells (Zhu et al., 2021). Other biomimetic biopolymers have also been such as peptide biopolymers showing advantages over coated tissue culture plastics for organoids generation. For instance, collagen-like peptide (CLP) with an integrin-binding motif arginine-glycine-aspartate conjugated to polyethylene glycol molecular templates (PEG-CLP-RGD) have shown capacity to help cerebellar cells self -assemble into organoid-like structures through its potential in mimicking ECM action (Balion et al., 2020).

#### 4.2.3 Organ-on-a-chip models

**FIGURE 3:**
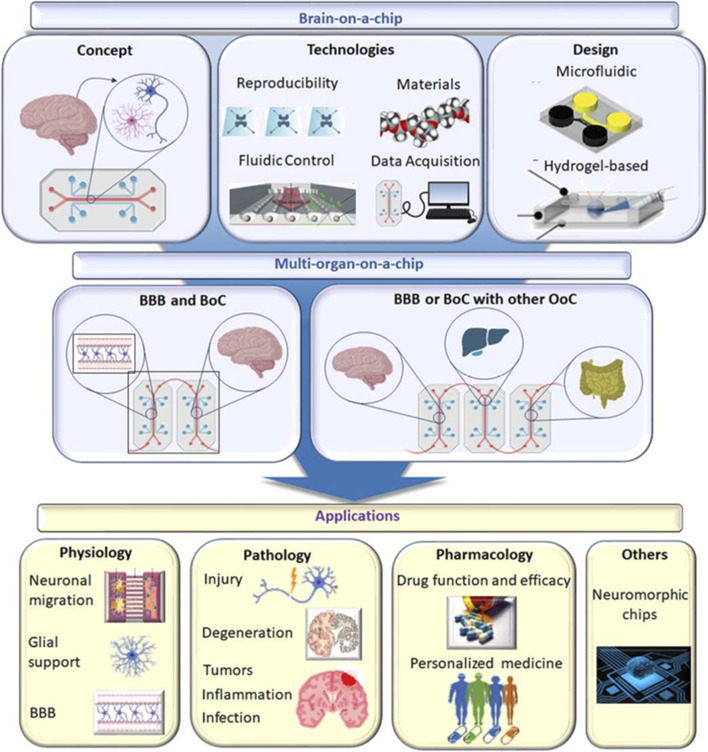
Overview of brain-on-a-chip platforms, including core design elements (microfluidics, materials, fluidic control), integration with multi-organ systems, and applications in physiology, pathology, and pharmacology. This schematic highlights the diversity and functional scope of microfluidic brain models used for studying neurological diseases and therapeutic responses *in vitro*. *Reproduced from* Amirifar et al. (2022), Figure 1, licensed under the Creative Commons Attribution-NonCommercial-NoDerivatives (CC BY-NC-ND) license: https://creativecommons.org/licenses/by-nc-nd/4.0/.

Organ-on-a-chip (OoC) technology leverages microfluidic systems to replicate the structural and functional characteristics of human organs *in vitro* (Figure 3). These platforms use micro-scale, fluid-filled channels lined with living cells to mimic tissue-tissue interfaces, dynamic fluid flow, and mechanical stimuli under physiologically relevant conditions (Srivastava et al., 2024). Among these, brain-on-a-chip models have shown great promise in recapitulating key aspects of neural physiology, including the blood-brain barrier (BBB), synaptic connectivity, and electrophysiological activity (Amirifar et al., 2022). OoC systems primarily utilize human-derived cells, especially induced pluripotent stem cell (iPSC)-derived neurons, astrocytes, and endothelial cells, to model both healthy and disease states (Vatine et al., 2019). However, early-stage models often employed rodent-derived cells—notably rat neurons, astrocytes, and endothelial cells—due to their wide availability and the robustness of established culture protocols. For instance, Adriani et al. (2017) developed a neurovascular model that co-cultured rat astrocytes and neurons with human endothelial cells, successfully reconstructing key aspects of the BBB in a hybrid system.

Brain-on-a-chip platforms have been extensively applied to model neurological disorders such as Alzheimer’s disease, Parkinson’s disease, and ischemic stroke (Cerutti and Brofiga, 2024). These systems have demonstrated the capacity to replicate hallmark pathological features, including amyloid-beta accumulation, tau hyperphosphorylation, and neuroinflammatory responses. Notably, Park et al. (2018) developed a 3D human brain-on-a-chip model that captured these key signatures of Alzheimer’s disease, thereby underscoring the platform’s potential in elucidating disease mechanisms and accelerating preclinical drug discovery. Compared to traditional 2D static cultures, brain-on-a-chip systems offer superior physiological relevance by replicating dynamic processes such as fluid flow, shear stress, and cell-cell interactions (Bhatia and Ingber, 2014). These platforms enable the real-time monitoring of critical parameters, including tissue barrier integrity (Douville et al., 2010) and cell migration dynamics (Nguyen et al., 2013). Additionally, the use of iPSC-derived human cell lines enhances the translational value of these models while reducing dependence on animal testing. Their capacity for live monitoring of cellular and barrier functions makes them particularly attractive for high-throughput drug screening and toxicological studies.

Despite their advantages, several challenges persist. Accurately reproducing the complexity of neural networks, including immune interactions, neurovascular coupling, and chronic disease phenotypes, remains difficult. The fabrication of microfluidic chips often requires specialized equipment and expertise, which can limit accessibility and scalability. Furthermore, maintaining long-term cell viability and functional stability within these platforms continues to be a significant obstacle (Castiglione et al., 2022).

Biomimetic scaffolds offer a promising avenue to address some of these limitations. For example, Roshanbinfar et al. (2025) developed crosslinked collagen–hyaluronan hydrogels that reduced matrix shrinkage while more closely emulating native extracellular matrix (ECM) properties. Their scaffolds supported the short-term viability of fibroblasts and endothelial cells, highlighting their suitability for soft tissue applications. While these materials have not yet been extensively applied to brain-on-a-chip systems, emerging evidence suggests they could significantly improve structural support, biocompatibility, and ECM mimicry in next-generation neural models.

## 5 Translation of biomimetic biopolymer composites into current healthcare

### 5.1 Current clinical needs for neural tissue engineering solutions

WHO set a global target by 2031 that “80% of countries will need to provide essential medicines and basic technologies required to manage NDs” (World Health Organization, 2023). It further recognises that, to attain this, better research tools are crucially needed (World Health Organization, 2023). Key UK Strategy documents further highlight that this will necessitate the facilitation of the development of better innovative materials that are cost-effective, accessible, sustainable and can have repurposing potential (Mathieson et al., 2020; 2021). In many ways as seen above biomimetic biopolymers may answer to these criteria. At this current stage, many of these materials are being researched and developed. We will explore the steps needed for their translation: manufacturing capabilities for cost effective scalability, the appropriate characterization and a roadmap towards effective translation.

### 5.2 Biomimetic biopolymer composites, advanced manufacturing and characterization

#### 5.2.1 Nanofabrication

Microfabrication encompasses techniques involving the synthesis of microstructures (see Table 1) and including the micron level. Techniques in microfabrication using biopolymers involve exploiting of biopolymer characteristics under certain conditions, such as pH, temperature and pressure, to produce desired microstructures (Moschakis and Biliaderis, 2017). Whilst production of nanoparticle incorporated scaffolds provides substrates for drug release, or suitable surfaces to promote nerve cell adhesion, growth, and proliferation, materials which can interface with nerves can be designed using lithography and etching microfabrication techniques (Zhai, 2024). Lithography and etching (nanoimprint lithography) have been used to create surfaces which mimic neurological relevant environments to be suitable for neuronal growth, and to create biosensor microelectrode arrays suitable of record (Fruncillo et al., 2021; Tran and Nguyen, 2017).

**TABLE 1:** Nanofabrication techniques for biomimetic biopolymers neural cell scaffolds.

Techniques	Summary	Biopolymers	Advantages	Disadvantages	Size and shape	References
Desolvation	Reduction of polymer solubility resulting in precipitation	Proteins	One pot reaction, adaptable by changing concentration of reactants	Requires addition ethanol of crosslinking agent	Nanoparticles 100 nm	Jahanban-Esfahlan et al. (2016), Hong et al. (2020), Arun et al. (2021), Meng et al. (2022)
Emulsification	Mixing of two organic and aqueous immiscible phases	Polysaccharide, Proteins	Easy formation of particles and agitation prevents aggregation	Shape limited to spheres, with limited control over size	Nanoparticles 100 nm	El-Sherbiny and Yacoub (2013), Jain et al. (2018), Lindemann et al. (2020)
Nano/ Electrospraying	Droplet drying of liquid into powder/with high voltage	Polysaccharide	Quick production at manageable temperatures, without potentially compromising biopolymer structural integrity	Low throughput in certain spraying modes	Nanoparticles 2 μm–200 nm	Mladenovska et al. (2007), Ranganath et al. (2009), Sable et al. (2024)
Coacervation	Exploiting pH dependent electrostatic interactions	Proteins	Simple preparation, produces materials of high stability and mechanical strength	Limited control over produced structure, including aggregation	0.05–1 μm	Clarke et al. (2006), Nigen et al. (2007), Moschakis and Biliaderis (2017), Mirlohi and Blocher McTigue (2025)
Ionic gelation	Electrostatic interactions between charged polysaccharides by addition of multivalent cations	Polysaccharides	Technique carried out at favorable conditions	Polymers must be ionic	Parameter determined	Fazil et al. (2012), Elnaggar et al. (2015), Lin et al. (2020), Kandil et al. (2021)
Electrospinning	Controlled fiber distribution from syringe pump with electric field applied	Polysaccharides	Variable in pore size, fiber length and diameter	Requires precise alignment and orientation, high voltage and clogged syringe tip	Nanofibers Diameter 100 nm	Wilk and Benko (2021), Luttge (2022)
Electrophoretic deposition	Assembly of charged particles into layers onto electrodes by application of electrical field	Polysaccharides	Generates uniform layers of desired thickness, including co-deposition	Deposition rate reduces over time, cannot be used on non-charged particles/molecules	Layers of thickness between hundreds of nanometers and tens of micrometers	Mishra et al. (2010), Gou et al. (2012), Zheng et al. (2014), Cheng et al. (2023)
Spin coating	Biopolymer solution mixed with substrate at high speed for homogenous distribution of biopolymer	Polysaccharides	Rapid process with the ability to alter composition during process	Expensive equipment, narrow range of viscosity obtainable, and clogged syringe tip	Coating thickness determined by speed	Vozzi et al. (2003), Song et al. (2020)
Lithography	Light sensitive substrate exposure to patterned light, solubilizing part of the substrate, which is dissolved, leaving the desired pattern	Silicon wafer or borosilicate glass, gold, biopolymer coatings	Accurate design of materials, including ability to use multiple different materials, low cost and high resolution	Leakage of materials reduced resolution and specificity	micro- and nano-scales creation of features <10 nm	Zheng et al. (2014), Traub et al. (2016), Yang et al. (2016), Szostak et al. (2017), Yu et al. (2020)
Etching and deposition	Often follows lithography, which allows removal of unwanted material and creation of distinct patterns. Thermal, chemical, electrolytic, ion, plasma, or combination of methods of etching	Removal of substrate	Can create specific shapes, for example aligning axons	Leakage of materials reduced resolution and specificity	micro- and nano-scales	Chinn, 2015; Yu et al. (2020), Kang et al. (2022)

#### 5.2.2 Nanotopographies

Numerous studies have explored the fabrication of nanoengineered surface topographies for various applications, including neural cell culture, adhesion, differentiation, stimulation and proliferation (Cho et al., 2024). These topographies can be randomly generated such as the roughened surfaces produced with wet chemistry by Brunetti et al. (2010) to investigate the response of human neuroblastoma cell line. Alternatively, they can also be designed as arrays of deterministic 3D features, such as tubes, pillars, or grooves. In this case, nanoscale fabrication techniques are employed. For example, e-beam lithography and deep reactive ion etching processes were utilized by Chen et al. (2020b) to generate Si nanoscale tubes for the subsequent study of GPE86 cells behaviour on such surfaces. To investigate the neural differentiation of rbMSCs cells, Zhang et al. (2019) used e-beam lithography and inductively coupled plasma (ICP) etching to pattern nanoscale grooves on a Si master before duplicating these features onto PVDF and PVC substrates via solvent-assisted molding. In a similar fashion, Yang et al. (2017) applied standard photolithography to produce a Si master with nanoscale grooves before their subsequent replication onto a PUA substrate via microtransfer molding. This substrate was eventually coated with a Ti film to examine the differentiation and electrophysiological maturation of human neural stem cells.

The ability to design and manufacture biophysical cues that mimic the nanoscale topography of native extracellular matrices remains essential for studying cellular behaviors. Currently, most nanoscale fabrication techniques rely on photolithography or scanning beam lithography approaches, typically performed in vacuum environments. This is not surprising given the widespread use of these processes in semiconductor and MEMS/NEMS manufacturing. However, to advance neural disease models and therapeutic platforms, alternative nanoscale fabrication methods should also be explored not only to expand the range of available substrate materials but also to help reduce the energy, resources and waste typically associated with conventional nanoscale fabrication techniques (Krishnan et al., 2008; Fang et al., 2017). One promising candidate in this context could be AFM tip-based nanomachining, especially for prototyping nanostructures. This method simply relies on the mechanical interaction between the tip of an AFM probe and a workpiece in a similar way to standard AFM imaging in contact mode, albeit with applied loads which are large enough to generate plastic deformation of the substrate (Yan et al., 2015; Xue et al., 2023). Compared with photolithography-based nanoscale pattern transfer techniques, AFM tip-based nanomachining does not require multiple process steps or a suite of highly specialised equipment while being also relatively cost effective. Thus, the technique is potentially accessible to a large number of research laboratories and straight-forward to implement on a wide range of substrates even for practitioners who only have basic AFM experience.

#### 5.2.3 Electrospinning

Electrospinning is a well-established nanofabrication technique used to produce highly porous, fibrous constructs that closely resemble the extracellular matrix (ECM) of native tissues (Jun et al., 2018). The process involves applying a high-voltage electric field to a polymer solution, which forms a charged jet that elongates into a Taylor cone (Abdulhussain et al., 2023). As the solvent evaporates, solidified fibers are deposited onto a grounded collector, forming a fibrous scaffold (Soliman et al., 2011).

One of the most compelling features of electrospun scaffolds for neural tissue engineering is their capacity for uniaxial fiber alignment (Xie et al., 2014), which introduces anisotropic physical cues that direct neuronal growth. Nutt et al., 2025 demonstrated that aligned polycaprolactone (PCL) nanofibers significantly enhanced the elongation and alignment of both astrocytes and neurons in primary cortical cultures compared to randomly oriented fibers and conventional flat substrates (Nutt et al., 2025). By facilitating a more biomimetic and spatially organized cellular architecture, electrospun constructs significantly improve the physiological relevance of neural models (Amores de Sousa et al., 2020).

Electrospinning is scalable, cost-effective and compatible with a broad spectrum of biopolymers (Wilk and Benko, 2021), allowing the design of scaffolds that better replicate the biochemical complexity of native neural ECM Mungenast et al., 2023). For example, Licciardello et al., 2024 incorporated polyaniline into a PCL-based matrix to produce electroconductive nanofibers, which enhanced the differentiation of neural progenitor cells into mature neurons, especially on aligned scaffolds (Licciardello et al., 2024). Further functionalization can be achieved through coaxial electrospinning (Khan et al., 2024), which enables the formation of core–shell fiber structures with multifunctional capabilities. Wu et al. (2018) used this approach to encapsulate PC12 cells within a polyvinyl alcohol (PVA) core surrounded by a PCL shell (Wu et al., 2018). Subsequent selective dissolution of the PVA core yielded hollow fibers containing cells, offering both structural guidance and spatial support for network formation in 3D neural cultures.

However, electrospinning also has limitations. Fiber morphology is highly sensitive to factors such as polymer viscosity, electrical conductivity and ambient conditions (Szewczyk and Stachewicz, 2020). Additionally, the use of organic solvents poses safety and sustainability concerns (Mosher et al., 2021), particularly in settings without advanced fume extraction systems. These challenges must be addressed to ensure reproducibility and safety in broader applications. Despite its limitations, electrospinning remains a powerful and versatile platform for fabricating scaffolds that replicate key biophysical and biochemical, aspects of the neural ECM (Mao et al., 2023). The capacity to guide neuronal development, support 3D culture and incorporate conductive and bioactive elements makes electrospinning an invaluable tool for modelling neural regeneration, injury and disease *in vitro* (Liu Z. H. et al., 2022; Palanisamy et al., 2025).

#### 5.2.4 Bioprinting

Unlike traditional scaffold-based models, 3D bioprinting offers significant advantages in the development of complex neural disease models by enabling the precise replication of brain tissue architecture and nerve-like fiber structures. This technology facilitates the study of neurodegenerative diseases, drug screening, and intricate cell–cell or cell–extracellular matrix (ECM) interactions, as well as cellular morphology. Moreover, 3D bioprinting supports the generation of patient-specific models with high reproducibility and scalability. It allows for the incorporation of diverse cell types, including organoids, spheroids, macrophages, and patient-derived cells. Bioprinting techniques vary based on material properties and include inkjet-based, extrusion-based, and light-assisted methods. These approaches support the use of composite bioinks and various crosslinking strategies such as chemical, dynamic, and photo-crosslinking. Commonly used bioink composite materials include alginate, gelatin, hyaluronic acid, silk fibroin, collagen, decellularized extracellular matrix (dECM), fibrinogen often combined with bioactive molecules and peptides.

In one study focused on spinal cord injury (SCI) repair, gelatin methacrylate (GelMA) and methacrylated hyaluronic acid (HAMA) were used to bioprint living nerve-like fibers embedded with neural stem cells The constructs were evaluated in a 4 mm-long complete spinal cord transection model in rats, resulting in enhanced neural circuit reconstruction and improved motor function recovery. This highlights the potential of 3D bioprinting to generate anatomically relevant and therapeutically effective constructs for SCI treatment (Yang et al., 2023). In another SCI-related study, a bioink composed of GelMA and oxidized hyaluronic acid, combined with N-cadherin mimetic peptide (HAVDI) and BDNF mimetic peptide (RGI), enhanced neural stem cell (NSC) mechanosensing. This improved cellular integration and led to significant motor and sensory recovery in SCI rats (Yang et al., 2025). Additionally, a composite bioink consisting of GelMA, Geltrex™, and RGD peptides supported neural cell survival and differentiation. Interestingly, this formulation promoted the de-differentiation of murine astrocytes into neural stem cells, indicating its potential for neural regeneration applications (Cruz et al., 2023).

To study cell morphology and cell–cell interactions, an ECM-mimetic hydrogel was developed using hyaluronic acid–PEG. The HA backbone enabled conjugation of adhesion peptides such as RGD and IKVAV. When used with fetal primary astrocytes (FPAs), the bioink enhanced cell–cell communication and network formation (Matthiesen et al., 2023). In another approach aimed at mimicking brain tissue mechanics, a bioink composed of sodium alginate, gelatin, and fibrinogen was engineered to match the elastic modulus of neural tissue. This formulation supported cellular physiological functions and enabled the fabrication of implants with personalized structures and multicellular distribution patterns (Gai et al., 2025).

#### 5.2.5 Casting, molding and gas foaming

**FIGURE 4:**
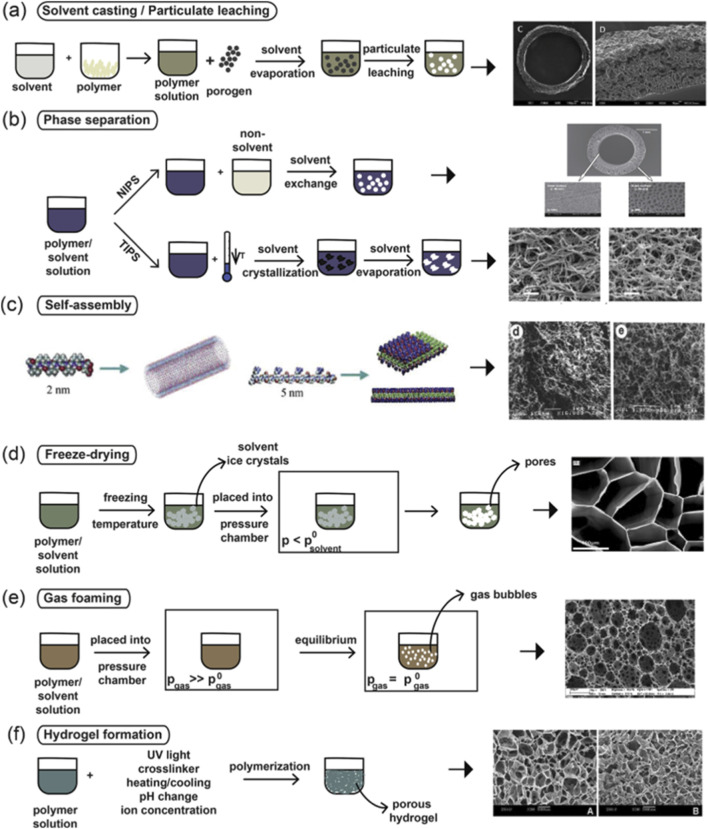
Schematic overview of common scaffold fabrication techniques in neural tissue engineering, including **(a)** solvent casting, **(b)** phase separation, **(c)** self-assembly, **(d)** freeze drying, **(e)** gas foaming, and **(f)** hydrogel formation. The figure highlights the stepwise inputs and resulting structural outcomes, providing visual insight into scaffold morphology and pore formation. Adapted from Papadimitriou et al. (2020, Figure 2), licensed under the Creative Commons Attribution 4.0 International License (CC BY 4.0): https://creativecommons.org/licenses/by/4.0/.

Casting, molding, and gas foaming are well-established scaffold fabrication techniques employed to produce customizable, three-dimensional (3D), porous biopolymer scaffolds that emulate the extracellular matrix (ECM) (Figure 4). In casting and molding, a liquid biopolymer precursor, such as gelatin or collagen, is introduced into a mold and solidified into a defined architecture (Mironov et al., 2008; Czepiel et al., 2023). Conversely, gas foaming relies on the incorporation of gas bubbles, either through physical or chemical processes, to generate porous structures without the use of organic solvents (Costantini and Barbetta, 2018). These techniques are widely recognized for their low cost, versatility, and ability to tune porosity for specific applications.

These fabrication methods are increasingly being applied to neural tissue engineering due to their potential to produce scaffolds that mimic key properties of neural ECM. For instance, gas-foamed PLA/silk fibroin scaffolds were shown to enhance Schwann cell proliferation, axonal regeneration, and functional recovery *in vivo,* attributed to their interconnected porous structure which facilitates cell infiltration and nutrient transport (Rao et al., 2019).

Sol-gel casting, a variant technique, allows for the use of metal alkoxide precursors to form biocompatible wet gels. These can be cast into desired shapes and thermally treated at low temperatures to produce either dense or porous structures, offering potential integration with bioactive materials for enhanced neural repair (Jarkov and Bowen, 2022). Such ECM-like scaffolds are particularly valuable for guiding cell behaviour and supporting regeneration in central and peripheral nervous system applications.

These fabrication methods offer several benefits:

- Gas foaming is notably solvent-free, making it environmentally friendly and suitable for applications sensitive to chemical residues (Zhou et al., 2024). It enables the creation of highly porous scaffolds (Arif et al., 2023), but challenges persist in achieving consistent pore interconnectivity and precise pore size control (Jahani et al., 2020).
- Solvent casting and particulate leaching are straightforward and replicable, with controllable porosity. However, these methods may suffer from residual solvent retention and are typically limited to thin film constructs (Eltom et al., 2019)
- Melt molding eliminates the need for solvents and provides good morphological control (Santos-Rosales et al., 2020), yet it requires elevated temperatures, which can degrade thermally sensitive biomolecules and cells (Allaf, 2018).

In summary, casting, molding, and gas foaming remain cost-effective, scalable, and accessible fabrication methods for neural tissue engineering. However, each presents unique trade-offs in terms of structural precision, sustainability, and integration of bioactivity, necessitating careful selection and optimization based on the specific application and material system.

#### 5.2.6 Electrophoretic deposition

Electrophoretic deposition (EPD) is a useful method which has been used for assembling biopolymers using electrical signals to form film coatings. This technique involves the movement of charged particles towards an electrode (either anode or cathode) using an electrical field, resulting in deposition of particles on electrode surface (Mishra et al., 2010). Using this technique, the film coatings can be produced to a desirable thickness by altering electrical current applied to the suspension containing conductive biopolymer, factors also influencing deposition rate during overall deposition time (Zheng et al., 2014). Although usually an organic solvent, using water as a solvent results in the formation of H_2_ and O_2_ gases at the electrodes, especially at high voltages, and as such, palladium electrodes have been used to absorb H_2_, along with strategies to reduce the voltage (Besra and Liu, 2007; Chávez-Valdez and Boccaccini, 2012). Biomimetic biopolymer structures generated by EPD upon electrodes produce porous materials, allowing for greater surface area for neural cell attachment and ingrowth (Seuss and Boccaccini, 2013).

Strategies using EPD for medical applications include forming a chitosan hydrogel membrane. In order to create films, membranes and coatings of chitosan, chitosan requires deprotonation using high pH conditions, as at basic conditions chitosan is uncharged (Li et al., 2017). This technique has been expanded to produce composite coatings of chitosan with molybdenum dioxide nanoparticles (Oliveira et al., 2021), and chitosan and curcumin (Karpiński et al., 2024). By alternating experimental parameters hierarchical structures and orientations of biopolymer upon the electrodes can be generated by alternating the electric field, rotating the biopolymers, with the potential of creating (Kasuga et al., 2022). Co-deposition to produce composite coatings hold promise as these coatings can have improved properties, making them more biodegradable and biocompatible, and more suitable materials for medical applications, along with being able to include therapeutic agents (Cheng et al., 2023).

**FIGURE 5:**
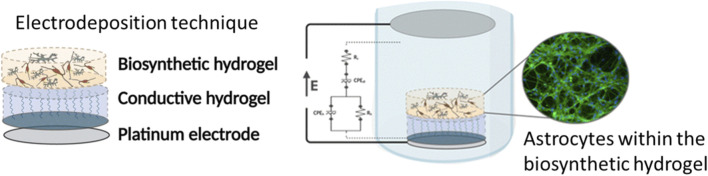
Generation of biomimetic biopolymeric electrodeposited material using conductive hydrogel layer for astrocyte encapsulation (Adapted from Boulingre et al. (2025) licensed under the Creative Commons Attribution 4.0 International License (CC BY 4.0): https://creativecommons.org/licenses/by/4.0/)ivecommons.org/licenses/by/4.0/).

By combining both a conductive hydrogel and biosynthetic hydrogel upon a platinum (Pt) electrode (Figure 5). In this example, EPD is used to stack layers onto a Pt electrode, the conductive hydrogel poly (3,4-ethylenedioxythiophene)/sodium *p*-toluenesulfonate layer, followed by the PVA-gelatin functionalized with norbornene. The generated biomimetic biopolymer structures supported growth and proliferation of astrocytes, by successful electrical stimuli reaching the biosynthetic hydrogel through the conductive hydrogel (Boulingre et al., 2025).

All the manufacturing and fabrication techniques described above also necessitate comprehensive characterization adequate for the development of biomimetic biopolymers composites. In an attempt to be comprehensive, we included a discussion on adequate biomimetic biopolymers characterization in Supplementary Appendix 1; Supplementary Material.

### 5.3 Efficiency validation *in vitro*, *in vivo* and in human trials

When thinking about adequate validation of biomimetic biopolymer composites for neural tissue engineering solutions in biological systems (*in vitro*/*in vivo* and in clinical settings) there are two perspectives to consider: (1) their own validation for therapeutic and clinical translational potential (2) their use as rigorous neural tissue models for validating other therapies and clinical or translational applications, which at the moment are heavily debated.

Validating biomimetic, biopolymer-based composites across *in vitro*, *in vivo*, and clinical settings is critical to ensuring their translational potential and therapeutic effectiveness as neural disease models. While *in vivo* animal models have long been the gold standard for evaluating biocompatibility, neuroregeneration, and device integration. They come with significant limitations, including ethical concerns, high costs and interspecies differences that can reduce clinical relevance. In response, there is a growing shift toward advanced *in vitro* systems such as 3D neural organoid, organ-on-chip platforms and bioengineered neural tissues (Rouleau et al., 2020).

This has enabled the fast-tracking of both the validation of biomimetic materials as potential therapeutic solutions and their integration into these advanced *in vitro* systems for testing other therapies. Such platforms increasingly succeed in mimicking critical features of the central nervous system, including the blood–brain, synaptic function and neuroinflammatory responses. This integration has enabled the obtention of information that was once available only via animal models and enabled predictions that heavily reduced the number of animal samples required and potentially reduced clinical risks ahead of clinical trials (Li et al., 2020). Such achievements can therefore help reduce research and development costs and, consequently, enable the improvement of access to neurotechnologies, including in low resource settings. When considering clinical translation of these technologies whether in research settings or clinical settings, several challenges lay ahead. For instance, their further expansion will only be enabled if accompanied by global regulatory integration and acknowledgement of their validation potential. Additionally, a more diverse clinical testing strategy needs to be employed to include low- and middle-income areas and vulnerable populations (women, elderly, ethnic global minorities). This is key for adequate translation as these demographics are the most affected by neurological diseases and lack of contextual data is often lacking in these situations, hindering application of these technologies in these contexts (World Health Organisation, 2010; Matshabane, 2021; World Health Organization, 2023).

### 5.4 Future perspectives – facilitating commercialization and regulation

As the development of biomimetic biopolymer-based composites accelerates in neural tissue engineering and therapeutics regulatory clarity regarding their classification and approvals pathways has become a critical concern. These materials often reside at the intersection of medical devices, biologics, and combination products, making classification complex and variable across jurisdictions. Current regulatory systems must adapt to the evolving nature of these multifunctional materials, particularly as they become more integrated into *in vitro* disease modelling and therapeutic strategies for neural disorders.

In most regions, biomaterials intended for neural applications are classified based on their intended use, duration of contact, and degree of invasiveness. Under the U.S. FDA framework, such materials often fall into Class II or Class III device categories requiring 510(k) clearance or premarket approval (PMA), respectively. Similarly, the European Union’s Medical Device Regulation (MDR) classifies these materials based on risk, with increasing scrutiny for implantable and long-term neural applications (FDA, 2025). However, classification is further complicated by the incorporation of biological agents, drug-eluting capabilities, or dynamic smart material properties, which can reclassify the product under combination or advanced therapy categories. Emerging regulatory frameworks globally, such as those being refined by China’s NMPA. India’s CDSCO, and other regional bodies, are beginning to address these complexities.

We recognize that while the path towards obtaining regulatory approval might be relatively mapped, the pathway towards adoption of neural technologies in clinical settings are a bit more challenging. This is because technologies adoption is governed by several factors such as government programs, healthcare budgets, reimbursements levels, costs benefits, public health priorities amongst others (Department of Healthcare and Social Services - UK, 2023). In regard to neurological disorders, a review of their prioritization in global and national priorities is necessary to put them in the forefront to improve access to neurological devices in practice (Mathieson et al., 2020; World Health Organization, 2023). While several countries pledged to make essential medications and essential technologies for neurological disorders accessible in primary care by 2031, efforts towards its realization are still required (World Health Organization, 2023). With the current unprecedented financial crisis in healthcare, increase in levels of financial and health disparities across countries, natural disasters and high costs of devices, implementation of such vision is becoming more challenging and yet even more necessary (World Health Organisation, 2010; World Health Organization, 2023).

Biomimetic biopolymer composites are therefore well-positioned in terms of biomaterials candidates for developing neural tissue engineering solution for sustainable and accessible healthcare due to several factors such as cost-efficiency, natural local abundance and sustainability, as seen previously. In fact, governments are starting to establish programs for adoption of novel technologies in national healthcare that embody principles of sustainability, patient safety and circularity which match what integrating biomimetic biopolymers composites can bring future innovative neural tissue engineering solutions (Department of Health and Social Care - UK, 2025). For instance, the UK government aims to start adopting the Design for Life Roadmap in healthcare to improve medical technologies’ accessibility, affordability and efficiency by establishing principles of circularity (e.g. repurposing, reusing), resilience and patient safety (Department of Health and Social Care - UK, 2025).

**FIGURE 6:**
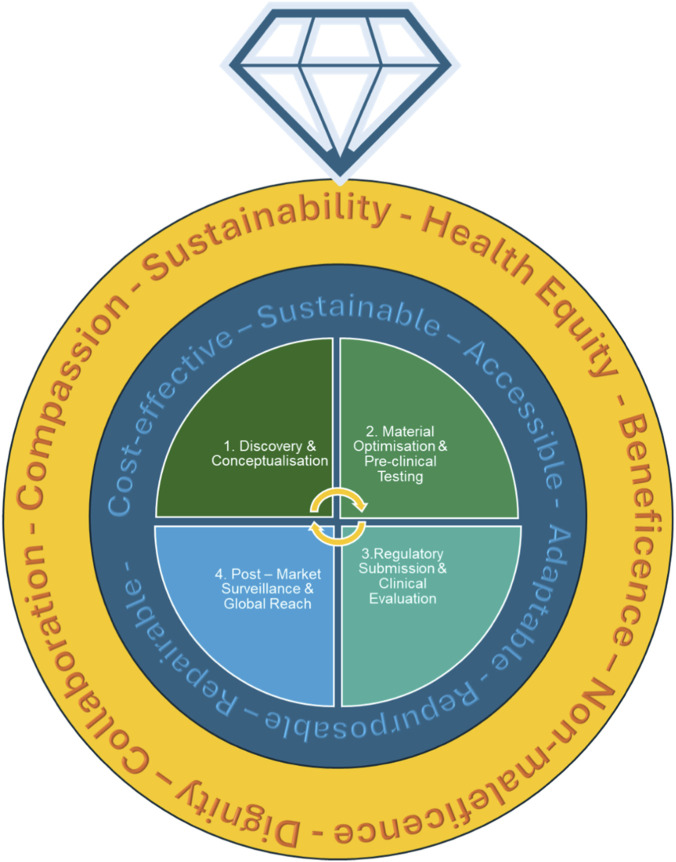
Schematic Roadmap for Accessible and Sustainable Biomimetic Biopolymers Research, Development and Translation. Inner circle: represents the key Development stages. No timeframe is included as the timeframes will be different for biomimetic biopolymer materials used for lab research purposes (e.g. tissue modelling or testing) and those for clinical purposes (e.g. Therapy). *Discovery and Conceptualization*: to clearly define unmet clinical needs by identifying biopolymer candidates, integrate design principles and develop intended functions. *Material optimization and Pre-clinical Testing*: to validate biofunctionality and align with emerging ethical policies. Regulatory Submission and Clinical Evaluation: to achieve regulatory clearance and perform clinical trials and compliance. Post-Market Surveillance and Global Reach: to monitor long-term performance of these biomimetic biopolymer materials and ensure global accessibility. Middle circle: represents the characteristics required by biomimetic materials for maximizing their accessibility, routine use in healthcare and adoption by healthcare systems as discussed previously. Some of these characteristics have been inspired by the UK Design for Life Roadmap (Department of Health and Social Care, 2024). Outer circle: illustrates core framework for development should be embedded in core ethical principles of sustainability, health equity, compassionate care and other principles derived from compassionate medical care: e.g. beneficence, non-maleficence (Bronzino and Peterson, 2006; Watts et al., 2023). Diamond merged with yellow outer circle: reflects the analogy of the engagement ring to highlight the necessity for renewed engagement to commit to these values and ethical principles to enable equitable access to biomimetic biopolymer technologies in healthcare through various mechanisms, for example as signatories as WHO member states (World Health Organization, 2023; World Health Organisation, 2025).

Additionally, the high level of disparities in neurological disorders, especially across low resource areas and vulnerable people, is currently stirring neuroethical principles to converge toward compassionate ethical principles as a viable way of resolving healthcare disparities and increase accessibility of novel neural technologies, including neural tissue engineering solutions (World Health Organisation, 2010; World Health Organisation, 2024a; National Academies of Sciences, 2023). Addressing these challenges, hence, requires proactive policy evolution and guidance that not only clarifies classification and approval processes but also integrates ethical imperatives into the core of biomedical material development and regulation. Hence, Figure 6 proposes an innovative research development roadmap that integrates these new trends and factors to be considered while developing biomimetic biopolymer-based composites neural tissue technology to ensure their sustained future adoption, accessibility and impact.

Neural tissue engineering biopolymers are at the boundaries between devices, drugs, and combination products, requiring regulatory and mechanistic evolution. Their bioactive, degradable, and variable nature challenges traditional safety and manufacturing standards, while overlapping mechanisms of action and the neural system’s sensitivity elevate evidence requirements. Addressing these barriers will require field-specific standards for neuro-conductivity and mechanics, standardized neuro-compatibility assays, mechanistic profiling and modelling, improved manufacturing controls for consistency and innovation management. For instance, directions towards this can already be seen. For instance, the British Standards Institution has recently published an innovation management standard (BS EN ISO 56000 series) to help manage innovation better for emerging innovation growth (British Standard Institution, 2021). Equally critical are modernized regulatory pathways and key ethical frameworks derived from compassionate medical care tailored to such biomaterials and their targeted application e.g. beneficence, non-maleficence (Bronzino and Peterson, 2006; Watts et al., 2023) (Figure 6). This is to ensure the products also reflect these ethical principles to ensure compassionate medical care by both practitioners and researchers. Together, these advances can provide the clarity and infrastructure needed to safely translate next-generation neural biopolymers for clinical applications.

## 6 Conclusion

Advanced biomimetic biopolymers have emerged as viable accessible, sustainable, scalable and cost-effective alternatives for neural tissue engineering with application in neurological disorders research and therapy. Their capacity to create high-level scaffolds mimicking both healthy and pathological ECM via their biomimetic properties makes them strong candidates for overcoming the constraints presented by other biomaterials categories, such as sustainable sourcing and financial accessibility. We further understand that their potential for cost-effective scalability is very tangible due to their potential adaptability and usability via various manufacturing and characterization techniques. Despite the current necessity in addressing issues such as confidence in embedding in routine research, regulation and clinical translation, the various advantages seen in this review justify further research and development. An adaptative and collaborative roadmap can help facilitate this process and enable their use in addressing the pressing need to considerable increase global provision of essential technologies and medicine for neurological disorders by 2031 (World Health Organization, 2023).
