# Supplementary material for: Emerging biomimetic biopolymer-based composites: advancing accessible and sustainable neural disease models and therapeutics
Source: Front Bioeng Biotechnol. 2026 Feb 19;13:1720254. doi: 10.3389/fbioe.2025.1720254 (PMC12960564; doi:10.3389/fbioe.2025.1720254)
Supplement: Supplementary file 1 [file DataSheet1.pdf]

Emerging Biomimetic Biopolymer-based Composites: Advancing Accessible and Sustainable Neural Disease Models and Therapeutics

Daniela Duc[1*], Jacob Pattem[2], Benjamin Gambrill[1], Polina Prokopovich[1], Aybike Kocaturkmen[1], Matthew Church[2], Amber Mays[2], Akash Bedi[2], Emmanuel Brousseau[3] and Oommen Oommen[1]

[1]School of Pharmacy and Pharmaceutical Sciences, College of Biomedical and Life Sciences, Cardiff University, Cardiff, United Kingdom.

[2]School of Dentistry, College of Biomedical and Life Sciences, Cardiff University, Cardiff, United Kingdom.

[3]School of Engineering, Department of Mechanical and Medical Engineering, Cardiff University, Cardiff, United Kingdom.

*** Correspondence:**
Dr Daniela Duc
ducd@cardiff.ac.uk

Keywords: Biomimetic biopolymer composites, neural tissue engineering, sustainability, accessibility, neural models, therapeutics

Supplementary Information

Appendix 1. Biomimetic Biopolymers Composite Characterisation

In characterising materials for brain modelling and therapeutic applications, four principal pillars can be identified: mechanical, physicochemical, bioactivity, and structural characterisation. Each of these domains provides essential insights for model validation and functional performance.

- Mechanical Characterization: This involves quantifying material properties such as stiffness (Young's modulus), shear modulus, elasticity, viscoelasticity, and compressive/tensile strength. In the context of neural applications, mechanical cues are particularly influential as CNS cells are highly sensitive to their biomechanical environment which can drive cell fate and influence regenerative

- Physicochemical Characterization: This domain assesses properties such as chemical composition, surface chemistry, degradation kinetics, hydrophilicity/hydrophobicity, porosity, and surface charge. For brain models and therapeutics, these parameters are essential for ensuring material compatibility with neural and for understanding how surface and bulk properties affect cellular behaviour.

- Bioactivity Characterisation: This encompasses the evaluation of biological responses to the material including cell viability proliferation differentiation, immune responses gene expression and scaffold. Such assessments are fundamental to confirming biocompatibility, minimizing adverse effects, and ensuring that engineered systems truly recapitulate the biological behaviour of native tissue.

- Structural Characterization: This focuses on the analysis of micro- and nano-structural features, including pore size and distribution, fibre alignment surface and overall scaffold architecture. Proper structural design is critical for facilitating nutrient diffusion enabling appropriate cell-matrix interactions and achieving accurate macro- and microscale mimicry of brain tissue.

Table 1. Summary of Biomimetic Biopolymers Composite Characterisation Techniques

Class of test	test	What it does	Relevance to neuro models	strengths	limits	References
**Mechanical**	Nano-indentation	Measures hardness and elastic modulus at micro and nano scale Forms force displacement curves	Evaluates stiffness of ECM or scaffold relevant to neurons/brain tissue	High spatial resolution; measures localized mechanical properties	Can damage tissue due to sharp point measurement	(Navindaran et al., 2023)
	AFM	can show Youngs modulus between cell to cell/material interaction creep compliance force relaxation	Can probe local mechanical interactions between cells/materials	High resolution	Analysis of surface only	(Kamble et al., 2023)
	Rheometry	Measures bulk viscoelastic properties Shear elastic moduli	Useful for characterizing hydrogels or ECM mimics in 3D brain models	Good for soft/biological samples;	Bulk measurement / macroscale only Destructive Assumes material homogeneity and isotropic	(Canovic et al., 2016)
	Tensile testing	Gives the young's and shear modulus, yield strength, ultimate compression strength and poisons ratio	Evaluates mechanical integrity of scaffolds and membranes in tension	Determines material strain Provides a stress strain curve Highly repeatable and versatile (axis wise) simple	Material slippage or misalignment can reduce accuracy – being optimised Destructive	(Zwirner et al., 2025)
	compression	Gives the young's and shear modulus, yield strength, ultimate compression strength and poisons ratio	Evaluates bulk scaffold/model compression properties	Provides a stress strain curve Assesses soft tissue in realistic hydrated environments Simple	Surface irregularities can affect results/accuracy	(Rashid et al., 2012)

**Structural**	AFM (form of nanoindentation)	Shows surface properties/ structure	Surface roughness & morphology affecting cell attachment Can be used in conjunction with microscopes to form spatially correct more detailed images	High resolution	Analysis of surface only Slow/multiple sections needed for large areas	(Lin et al., 2013)
	Multiphoton microscopy	Deep 3D optical imaging of fluorescent samples through non-linear excitation of fluorophores	Visualizes deep brain regions or 3D scaffolds or organoids Can study morphogenesis and differentiation	Good depth penetration less photodamage Good for high scattering live tissue	Expensive. requires fluorophores, possible issues with bulk heating	(Lacin and Yildirim, 2024)
	Confocal laser scanning microscopy	Optical sectioning of fluorescent samples	Heps visualise the pore size/ architecture of 3D neural scaffolds	High resolution/clear image	Limited penetration depth Need to attach fluorescent probes/ have a fluorescent sample	(Tomlins et al., 2004)
	Fluorescence imaging/microscopy	fluorescent dyes/ proteins to label and visualize specific cellular structures/ molecules and their dynamics	visualization of neural cell morphology, protein expression, and connectivity in 2D and 3D neural models Can give mechanistic insights in real time	High specificity Live-cell imaging possible (in situ) Real time	Photobleaching Limited depth penetration May require fixation or staining Fluorophore/ probe dependent	(Yang and Cui, 2023)
	XRD	Shows size shape and crystalline structure Helps visualise tissue properties	Provides maps relevant to tissue properties Orientation of crystals	Good resolution at atomic scale Can measure internal stress and architecture	Limits of application Sample needs to be small Complex crystalline patterns make	(Carboni et al., 2017)

					analysis difficult	
	Swelling and porosity	Assesses poroelasticity and viscoelasticity	Important for determining viscoelastic properties of tissue and nutrient diffusion	Simple, important for models and viability	May damage tissue with overloading/ continual loading	(Su et al., 2023)
**Physiochemical**	FTIR	Shows bonding/chemical structure Bond lengths and angles, aggregate states, protein structures, H bonding	Can assess damage to tissue and molecular changes over time	Can be used at near native states without label manipulation quantitative	Requires complex interpretation Hard to detect small signals in a large background	(Surowka et al., 2020)
	XPS	Binding energy Quantifying elemental composition Chemical states Structural differences	Assesses surface chemistry for cell-material interaction	Sensitive and quantitative	Requires a vacuum	(De Carvalho et al., 2020)
	Raman Spectroscopy	Shows structural, chemical and electric properties Provides information on hydrodynamic size and size distribution	Can help provide information on tissue/model properties Can compare/ differentiate normal to pathological brain tissue	High specificity and sensitivity Minimal tissue pre Non-invasive	(limited) Spatial resolution to micrometres Analyses small cross sections Fluorescence can interfere with results	(Krzemińska et al., 2025)
	Optical clearing	Modifies the optical properties of biological samples by changing the/some components to a uniform RI	Allows better visualisation of spheroids/3D neural models Visualisation of deep brain	Can improve use of other techniques e.g. microscopy. Easy and low cost	May change density. May cause some sample loss or property change	(Costa et al., 2019)
	patch clamp-based electrophysiological recording	Measures ion channel activity by forming a tight seal between a glass micropipette	Key for studying neuronal excitement and function	Can assess single-channel activation and different potential (action,	Complex methodology / technically	(Kodirov, 2023)

		and the cell membrane.		junction, endplate, resting)		
	oxygen probe	phosphorescent dyes that change their phosphorescence lifetime in response to oxygen levels, can be cell penetrating	Important for studying oxygenation, growth and differentiation condition of individual cells in models/tissue	Improvement in some models, minimal effect on cell viability	Poor biodistribution and analytical performance	(Dmitriev et al., 2014)
**Biological**	Cell viability assays (live/dead/,MTT, alamar blue)	Measures metabolic activity or viability	Assesses cell health in the scaffold/model	Can use dyes that don't affect cells so preserves native function<br>Simple and fast	Dyes can be cytotoxic<br>Metabolism and viability aren't always interchangeable	(Whye et al., 2024)
	Proliferation assays (BrdU, Ki-67)	Tests for known markers of proliferation in supernatant of cells	Can help track cell health and growth rate in 3D scaffolds/neural models	Specific and can be quantitative	May need fixation<br>Requires immunodetection and robust/specific labelling	(Eminaga et al., 2016)
	Protein analysis (western blott, ELISA, Immunofluorescence)	Detects specific proteins/markers	Allows identification of cell type<br>Ensure proper differentiation	Good for analysis of cell markers<br>Specific<br>Often quantitative	Needs optimization, can be expensive	(Rietze and Reynolds, 2006)
	Gene expression (RT-qPCR, RNA-seq)	Detects cell surface marker and protein expression<br>Can generate transcriptomic profiles	Can help track maturation of cells<br>Can help identify and classify neural cells developed<br>Can help identify reference genes	Highly sensitive high-throughput accessible	Requires RNA isolation<br>Needs bioinformatic analysis<br>Can be destructive	(Liu et al., 2023)
	degradation	Measures breakdown of material over time<br>In vivo<br>Fluorescence over time<br>in vitro	Evaluates scaffold lifespan and biocompatibility<br>Important for compliance	Needed to fulfil safety regulations	Destructive<br>Hard to replicate in vivo condition (in vitro to in vivo translation)	(De Jong et al., 2020)

		done by mass loss	with safety regulati ons		Results vary (slower degradation in vivo than in vitro)	

References

Canovic, E. P., Qing, B., Mijailovic, A. S., Jagielska, A., Whitfield, M. J., Kelly, E., et al. (2016). Characterizing Multiscale Mechanical Properties of Brain Tissue Using Atomic Force Microscopy, Impact Indentation, and Rheometry. *J Vis Exp* 2016, 54201. doi: 10.3791/54201

Carboni, E., Nicolas, J.-D., Töpperwien, M., Stadelmann-Nessler, C., Lingor, P., and Salditt, T. (2017). Imaging of neuronal tissues by x-ray diffraction and x-ray fluorescence microscopy: evaluation of contrast and biomarkers for neurodegenerative diseases. *Biomed Opt Express* 8, 4331. doi: 10.1364/BOE.8.004331

Costa, E. C., Silva, D. N., Moreira, A. F., and Correia, I. J. (2019). Optical clearing methods: An overview of the techniques used for the imaging of 3D spheroids. *Biotechnol Bioeng* 116, 2742–2763. doi: 10.1002/BIT.27105

De Carvalho, A. G., Barnes, J.-P., Renault, O., Mariolle, D., Gaude, C., Ratel, D., et al. (2020). Combining surface-sensitive microscopies for analysis of biological tissues after neural device implantation. *Biointerphases* 15. doi: 10.1116/6.0000110

De Jong, W. H., Carraway, J. W., and Geertsma, R. E. (2020). In vivo and in vitro testing for the biological safety evaluation of biomaterials and medical devices. *Biocompatibility and Performance of Medical Devices*, 123–166. doi: 10.1016/B978-0-08-102643-4.00007-0

Dmitriev, R. I., Kondrashina, A. V., Koren, K., Klimant, I., Zhdanov, A. V., Pakan, J. M. P., et al. (2014). Small molecule phosphorescent probes for O2 imaging in 3D tissue models. *Biomater Sci* 2, 853–866. doi: 10.1039/C3BM60272A

Eminaga, S., Teekakirikul, P., Seidman, C. E., and Seidman, J. G. (2016). Detection of markers of cell proliferation by immunofluorescent staining and microscopy imaging in paraffin-embedded tissue sections. *Curr Protoc Mol Biol* 115, 14.25.1. doi: 10.1002/CPMB.13

Kamble, Y., Raj, A., and Thakur, A. (2023). Artificial Neural Network-Aided Computational Approach for Mechanophenotyping of Biological Cells Using Atomic Force Microscopy. *J Biomech Eng* 145. doi: 10.1115/1.4056916

Kodirov, S. A. (2023). Whole-cell patch-clamp recording and parameters. *Biophys Rev* 15, 257. doi: 10.1007/S12551-023-01055-8

Krzemińska, A., Czapiga, B., and Koźba-Gosztyła, M. (2025). Accuracy of Raman spectroscopy in discriminating normal brain tissue from brain tumor: A systematic review and meta-analysis. *Spectrochim Acta A Mol Biomol Spectrosc* 329, 125518. doi: 10.1016/J.SAA.2024.125518

Lacin, M. E., and Yildirim, M. (2024). Applications of multiphoton microscopy in imaging cerebral and retinal organoids. *Front Neurosci* 18, 1360482. doi: 10.3389/FNINS.2024.1360482/BIBTEX

Lin, P. C., Lin, S., Wang, P. C., and Sridhar, R. (2013). Techniques for physicochemical characterization of nanomaterials. *Biotechnol Adv* 32, 711. doi: 10.1016/J.BIOTECHADV.2013.11.006

Liu, D. D., He, J. Q., Sinha, R., Eastman, A. E., Toland, A. M., Morri, M., et al. (2023). Purification and characterization of human neural stem and progenitor cells. *Cell* 186, 1179-1194.e15. doi: 10.1016/j.cell.2023.02.017

Navindaran, K., Kang, J. S., and Moon, K. (2023). Techniques for characterizing mechanical properties of soft tissues. *J Mech Behav Biomed Mater* 138, 105575. doi: 10.1016/J.JMBBM.2022.105575

Rashid, B., Destrade, M., and Gilchrist, M. D. (2012). Determination of friction coefficient in unconfined compression of brain tissue. *J Mech Behav Biomed Mater* 14, 163–171. doi: 10.1016/J.JMBBM.2012.05.001

Rietze, R. L., and Reynolds, B. A. (2006). Neural Stem Cell Isolation and Characterization. *Methods Enzymol* 419, 3–23. doi: 10.1016/S0076-6879(06)19001-1

Su, L., Wang, M., Yin, J., Ti, F., Yang, J., Ma, C., et al. (2023). Distinguishing poroelasticity and viscoelasticity of brain tissue with time scale. *Acta Biomater* 155, 423–435. doi: 10.1016/j.actbio.2022.11.009

Surowka, A. D., Gianoncelli, A., Birarda, G., Sala, S., Cefarin, N., Matruglio, A., et al. (2020). Soft X-ray induced radiation damage in thin freeze-dried brain samples studied by FTIR microscopy. *J. Synchrotron Rad* 27, 1218–1226. doi: 10.1107/S1600577520010103

Tomlins, P., Grant, P., Mikhalovsky, S., James, S., and Mikhalovska, L. (2004). Measurement of Pore Size and Porosity of Tissue Scaffolds. *J ASTM Int* 1, 1–8. doi: 10.1520/JAI11510

Whye, D., Norabuena, E. M., Srinivasan, G. R., Wood, D., Polanco, T. J., Makhortova, N. R., et al. (2024). A Hybrid 2D-to-3D in vitro Differentiation Platform Improves Outcomes of Cerebral Cortical Organoid Generation in hiPSCs. *Curr Protoc* 4, e70022. doi: 10.1002/CPZ1.70022

Yang, Q., and Cui, X. T. (2023). Advanced in vivo fluorescence microscopy of neural electronic interface. *MRS Bull* 48, 506–517. doi: 10.1557/S43577-023-00530-7/FIGURES/5

Zwirner, J., Waddell, J. N., Ondruschka, B., and Li, K. C. (2025). 3D-printed suction clamps for tensile testing of brain tissue. *J Mech Behav Biomed Mater* 163, 106873. doi: 10.1016/J.JMBBM.2024.106873
